# A Metabolism-Related Gene Prognostic Index Bridging Metabolic Signatures and Antitumor Immune Cycling in Head and Neck Squamous Cell Carcinoma

**DOI:** 10.3389/fimmu.2022.857934

**Published:** 2022-06-30

**Authors:** Kunpeng Du, Jingwen Zou, Baiyao Wang, Chunshan Liu, Muhammad Khan, Tao Xie, Xiaoting Huang, Piao Shen, Yunhong Tian, Yawei Yuan

**Affiliations:** ^1^ Department of Radiation Oncology, Affiliated Cancer Hospital & Institute of Guangzhou Medical University, Guangzhou, China; ^2^ Department of Liver Surgery of the Sun Yat-sen University Cancer Center, Guangzhou, China

**Keywords:** metabolism, antitumor immune cycling, immunotherapy, prognosis, individualized precision therapy

## Abstract

**Background:**

In the era of immunotherapy, predictive or prognostic biomarkers for head and neck squamous cell carcinoma (HNSCC) are urgently needed. Metabolic reprogramming in the tumor microenvironment (TME) is a non-negligible reason for the low therapeutic response to immune checkpoint inhibitor (ICI) therapy. We aimed to construct a metabolism-related gene prognostic index (MRGPI) for HNSCC bridging metabolic characteristics and antitumor immune cycling and identified the immunophenotype, genetic alternations, potential targeted inhibitors, and the benefit of immunotherapy in MRGPI-defined subgroups of HNSCC.

**Methods:**

Based on The Cancer Genome Atlas (TCGA) HNSCC dataset (n = 502), metabolism-related hub genes were identified by the weighted gene co-expression network analysis (WGCNA). Seven genes were identified to construct the MRGPI by using the Cox regression method and validated with an HNSCC dataset (n = 270) from the Gene Expression Omnibus (GEO) database. Afterward, the prognostic value, metabolic activities, genetic alternations, gene set enrichment analysis (GSEA), immunophenotype, Connectivity map (cMAP), and benefit of immunotherapy in MRGPI-defined subgroups were analyzed.

**Results:**

The MRGPI was constructed based on *HPRT1*, *AGPAT4*, *AMY2B*, *ACADL*, *CKM*, *PLA2G2D*, and *ADA*. Patients in the low-MRGPI group had better overall survival than those in the high-MRGPI group, consistent with the results in the GEO cohort (cutoff value = 1.01). Patients with a low MRGPI score display lower metabolic activities and an active antitumor immunity status and more benefit from immunotherapy. In contrast, a higher MRGPI score was correlated with higher metabolic activities, more TP53 mutation rate, lower antitumor immunity ability, an immunosuppressive TME, and less benefit from immunotherapy.

**Conclusion:**

The MRGPI is a promising indicator to distinguish the prognosis, the metabolic, molecular, and immune phenotype, and the benefit from immunotherapy in HNSCC.

## Introduction

The large-scale application of immune checkpoint inhibitor (ICI) therapy, such as anti-programmed death 1 (PD-1), anti-programmed death-ligand 1 (PD-L1), and anti-cytotoxic T lymphocyte-associated antigen-4 (CTLA-4), has greatly improved the survival rate of cancer patients (1–7). In head and neck squamous cell carcinoma (HNSCC), especially in recurrent or metastatic HNSCC, ICI therapy has been approved as an effective treatment and achieved significant survival benefits (8–11). However, the polarized therapeutic response to ICI therapy between different patients is the major limitation of immunotherapy. There are several reasons why patients cannot benefit from immunotherapy, among which metabolic reprogramming in the tumor microenvironment (TME) is a non-negligible reason.

It has been recognized that the metabolic reprogramming of tumor cells characterized by the Warburg effect is an important hallmark of tumors and is considered a driver of cancer progression (12–14). Metabolic reprogramming provides an inherent advantage for tumors to compete for nutrition, survive, and proliferate in the unique hypoxic and acidotic TME (12, 15). For example, in the competition of TME components for nutrients, especially glucose, the Warburg effect enhances glycolysis or aerobic glycolysis of tumor cells under hypoxic conditions (12). However, other immune cells are at a disadvantage in the competition for nutrients in the hypoxic microenvironment because they mainly rely on oxidative phosphorylation (OXPHOS) for energy (16). Therefore, tumor cells outcompete T cells for glucose consumption and can metabolically restrict T cells, directly dampening their effector function and allowing tumor progression (12, 17). Interestingly, recent studies revealed that metabolic reprogramming also occurs in numerous immune cells within the TME and profoundly influences the trajectories of immune cell differentiation and fate, which impaired the functions of the immune cells (16, 18–22). For example, a recent study demonstrated that the TME can stimulate CD36 expression in intratumoral regulatory T (Treg) cells (a major immunosuppressive cell that blocks antitumor immunity and affects cancer immunotherapy) and then increase lipid metabolism in intratumoral Treg cells, which might support the adaptation to a lactate-rich TME by lipid metabolic programming of Treg cells (23). Numerous studies have reported that tumor cells produce a large amount of lactic acid through aerobic glycolysis and release the redundant lactic acid into the TME *via* monocarboxylate transporter 4 (MCT4) (24). It has been reported that lactic acid and acidification suppress tumor necrosis factor (TNF) secretion of human monocytes through glycolysis inhibition (25). Furthermore, tumor cell-derived lactate induces vascular endothelial growth factor (VEGF) expression and M2-like polarization of macrophages by stabilizing hypoxia-inducible factor 1α (HIF1α) (26). A recent study demonstrated that lactic acid can also induce M2-like gene activation in macrophages through a novel epigenetic modification, histone lactylation (27). Another study found that the TME induces tumor cells to produce retinoic acid (RA), which polarizes intratumoral monocyte differentiation toward tumor-associated macrophages (TAMs) and away from dendritic cells (DCs) *via* suppression of DC-promoting transcription factor Irf4 (28). To adapt to the low-glucose lactic acid-enriched TME, some immune cells also undergo metabolic reprogramming. The upregulated CD36 fine-tuned mitochondrial fitness *via* peroxisome proliferator-activated receptor-β signaling, programming Treg cells to adapt to a lactic acid-enriched TME (23, 29). Additionally, the Treg cell transcription factor Foxp3 reprograms T-cell metabolism to tolerate the low-glucose lactate-rich environments by suppressing Myc and glycolysis, enhancing OXPHOS, and increasing nicotinamide adenine dinucleotide oxidation (30). Moreover, increasing evidence suggested that the low response rate to immunotherapy of patients is presumably due to the exuberant energy from multiple metabolic sources in tumors and nutrient deprivation and metabolite accumulation in the TME, which limits the recovery of antitumor immunity (31). Therefore, identifying the unique metabolism-related molecular characteristics and TME landscape that easily benefit from ICI therapy has become a crucial proposition. There are currently plenty of prognostic markers for HNSCC, including single gene biomarkers or gene signatures composed of multiple genes. For example, Chen et al. (32) constructed a prognostic index composed of three immune genes, *SFRP4*, *CPXM1*, and *COL5A1*, which can well distinguish the prognosis and molecular and immune characteristics of HNSCC patients. A novel signature, consisting of seven ferroptosis-related genes, developed by He et al. (33) can also serve as a prognostic marker for predicting prognosis in HNSCC. Our previous study also found that the signature composed of *CNFN* and *DEPDC1* could serve as an independent biomarker to predict the risk of lymphovascular invasion and as a prognostic marker for HNSCC (34). However, the prognostic potential of the molecular characteristics of tumor metabolism for conventional therapy and immunotherapy in HNSCC remains to be fully explored.

In this study, we aim to find a metabolic index that could be used to identify HNSCC patients who can benefit from conventional treatment. More importantly, this index could reflect the immunogenic or immune activities of each key step in the antitumor immunity processes of different HNSCC patients so that could predict the prognosis of comprehensive therapy and immunotherapy. Thus, we used the weighted gene co-expression network analysis (WGCNA) to identify metabolism-related hub genes associated with the prognosis of HNSCC patients and used the Cox regression analysis to develop a metabolism-related gene prognostic index (MRGPI). The MRGPI consists of hypoxanthine guanine phosphoribosyl transferase 1 (*HPRT1*); 1-acylglycerol-3-phosphate O-acyltransferase 4 (*AGPAT4*); amylase alpha 2B (*AMY2B*); acyl-CoA dehydrogenase long-chain (*ACADL*); creatine kinase, muscle (*CKM*); phospholipase A2 Group IID (*PLA2G2D*); and adenosine deaminase (*ADA*) genes. Then, we profiled the metabolic and molecular characteristics, the status of seven-step antitumor immunity processes, and the TME landscape of the different MRGPI subgroups. Moreover, we examined the predictive ability of the MRGPI in the immunotherapy cohorts and compared it with other classic signatures that can predict the efficacy of immunotherapy, such as tumor inflammation signature (TIS) and tumor immune dysfunction and exclusion (TIDE). The results indicated that the MRGPI is a promising prognostic biomarker for patients undergoing conventional therapies and ICI immunotherapy.

## Materials and Methods

### Patients and Datasets

The latest RNA sequencing (RNA-seq) data, clinicopathologic information, and survival data of 546 HNSCC samples, including 502 tumor samples and 44 normal samples, were obtained from The Cancer Genome Atlas (TCGA) database through the Genomic Data Commons Data Portal (GDC; https://portal.gdc.cancer.gov/). RNA-seq data of 270 HNSCC tumor samples (GSE65858), corresponding survival information, and the Sequencing platform annotation information (GPL570) were downloaded from the Gene Expression Omnibus (GEO) database (https://www.ncbi.nlm.nih.gov/geo/). The list of metabolism-related genes was collected and integrated from the Kyoto Encyclopedia of Genes and Genomes (KEGG) metabolism-related pathways (https://www.kegg.jp/kegg/kegg1.html).

### Identification of Metabolism-Related Differentially Expressed Genes

To obtain the metabolism-related differentially expressed genes (DEGs), we first obtained the DEGs (adj. p < 0.05, |log2FC| >1.0) between the 502 tumor samples and 44 normal samples of HNSCC from TCGA-HNSCC project using the limma package of R software. Then, by intersecting with the metabolism-related gene lists, the differentially expressed metabolism-related genes were extracted from the abovementioned DEGs. Volcano plots of DEGs and differentially expressed metabolism-related genes were plotted using the ggplot2 package of R. The clusterProfiler package of R was used to perform Gene Ontology (GO) and KEGG analysis of the obtained metabolism-related DEGs to explore the biological functions and processes involved in these genes.

### Identification of Metabolism-Related Hub Genes

The WGCNA was used to recognize the metabolism-related hub genes (35). The first step was to calculate the correlation coefficient (Pearson coefficient) between any two genes and construct the similarity matrix. To measure whether two genes have similar expression patterns, correlation coefficient weighted values were used during WGCNA. Next, the best “soft threshold” was determined graphically as β = 4 to ensure a scale-free network distribution. The similarity matrix and β value were adopted to construct an adjacency matrix and then transformed into a topological matrix with the topological overlap measure (TOM) describing the degree of association between genes. Here, 1-TOM was used as the distance to cluster the genes, and then the dynamic pruning tree was constructed to generate the coexpression gene modules. Finally, the similar gene modules were determined by merging modules whose distance is less than 0.25. The correlation between each module and HNSCC was calculated by the eigengene function in the “WGCNA” R package. The most significantly related module was selected for the follow-up study. In this module, the edges between two genes with weight >0.2 were selected to plot the network. The genes in the network with a degree ranking in the top 50 were the nominated hub genes. The best cutoff value of the impact of each hub gene on overall survival (OS) was obtained through the Survminer package of R. Only genes significantly related to survival (p < 0.05, log-rank test) were considered as the metabolism-related hub genes.

### Development and Validation of the Metabolism-Related Gene Prognostic Index

Among the obtained metabolism-related hub genes, the genes significantly affecting OS were identified by univariate Cox regression analysis and then utilized to construct the MRGPI by multivariate Cox regression analysis. The MRGPI of each sample was calculated as follows: MRGPI = ,where represents the regression coefficient between the gene (i) and HNSCC prognosis, and represents the expression level of the gene (i).

The HNSCC patients from TCGA-HNSCC project (training cohort) were divided into the high-MRGPI group and low-MRGPI group according to the median MRGPI. The ability of the MRGPI to discriminate the prognosis of patients was respectively evaluated by the Kaplan–Meier survival curve and the log-rank test of TCGA and GEO cohorts (validation cohort).

In addition, to verify the independent prognostic role of the MRGPI, the MRGPI and other clinicopathological factors were included in univariate and multivariate Cox regression analyses.

### Identification of Metabolic Characteristics of the Different Metabolism-Related Gene Prognostic Index Subgroups

The gene set variation analysis (GSVA) package of R was used to calculate the common metabolic activity scores of each patient (36), including beta-alanine metabolism; fatty acid metabolism; glutathione metabolism; glycerolipid metabolism; nitrogen metabolism; purine metabolism; pyruvate metabolism; starch and sucrose metabolism; glutamate and glutamine metabolism; glycogen metabolism; glucose metabolism; and alanine, aspartate, and glutamate metabolism. Then, the difference in metabolic activities was compared between the high- and low-MRGPI groups.

Specifically, we firstly obtained the gene lists reflecting these metabolic activities (**Supplementary Table S1**) from the KEGG (https://www.kegg.jp/) and Reactome (https://reactome.org/) pathway databases, which are open-source, open-access, manually curated, and peer-reviewed pathway databases and gained wide acceptance (37, 38). Then, we used the method of Hänzelmann et al. (36) to assess the GSVA enrichment scores of metabolic activities in each patient: 1) Input RNA-seq counts and a list of metabolic gene sets; 2) Gene expression level statistic: Kernel estimation of the cumulative density function (kcdf); 3) Rank ordered for each sample by the expression-level statistic; 4) Calculated the Kolmogorov–Smirnov-like rank statistic for every gene set; 5) Different score distributions; 6) Output a matrix containing pathway enrichment scores for each gene set and sample. The detailed method is available as a Bioconductor package for R under the name GSVA at http://www.bioconductor.org.

### Identification of Molecular Characteristics of the Different Metabolism-Related Gene Prognostic Index Subgroups

To discover the molecular characteristics of the different MRGPI subgroups, we firstly used the limma package of R to find out the DEGs between the high- and low-MRGPI groups. Then, the gene set enrichment analysis (GSEA) was used to enrich the signal pathways involved in different MRGPI subgroups based on the KEGG gene sets (c2. cp. kegg. v7.4) by using the clusterProfiler package of R [p < 0.05, false discovery rate (FDR) <0.25].

The data of genetic alterations (simple nucleotide variation) of HNSCC was obtained from TCGA GDC data portal, and R’s Maftools package was used to analyze the number and categories of gene mutations in the two MRGPI subgroups. The tumor mutation burden (TMB) of each sample was also calculated by the Maftools package of R.

### Comprehensive Analysis of Immune Characteristics and Immune Checkpoint Inhibitor Therapy in the Different Metabolism-Related Gene Prognostic Index Subgroups

There are several anticancer immune steps in the antitumor immune cycle, including Release of cancer antigens, Cancer antigen presentation, Priming and activation, Trafficking of immune cells to tumors, Infiltration of immune cells into tumors, Recognition of cancer cells by T cells, and Killing of cancer cells. These 7 antitumor immune processes constitute the antitumor immune cycle. The tracking tumor immunophenotype (TIP) website collected and curated gene lists that represent these seven antitumor immune steps, which are presented in **Supplementary Table S2 (**, 39). Single-sample gene set enrichment analysis (ssGSEA) is a method that calculates the absolute enrichment degree of a given gene set based on these gene expression values of the sample sequencing data and then indirectly reflects the activity or enrichment degree of the given gene set (40). Therefore, we used the ssGSEA algorithm to score the seven antitumor immune processes during the antitumor immune cycle of 499 HNSCC tumor samples and to evaluate the tumor immunophenotype in high-MRGPI and low-MRGPI subgroups. Scores for each antitumor immune step for each HNSCC patient in this study were attached in **Supplementary Table S3**. To further evaluate the difference in the level of immune cell infiltration between different MRGPI score subgroups, we used the CIBERSORT algorithm to quantitatively analyze the relative abundance of 22 immune cells in 499 HNSCC tumor samples (41). The specific information on the proportion of each immune cell infiltration in each patient was presented in **Supplementary Table S4**.

To compare the immune state within the TME of the two MRGPI subgroups. The ESTIMATE score, immune score, and stromal score were calculated using the ESTIMATE algorithm. The ESTIMATE algorithm is a tool that uses gene expression data to predict tumor purity and the degree of infiltrating stromal/immune cells in tumor tissue. The ESTIMATE algorithm is based on ssGSEA and generates three scores: 1) stromal score (capturing the presence of stroma in tumor tissue), 2) immune score (representing the infiltration of immune cells in tumor tissue), and 3) ESTIMATE score (inferred tumor purity).

Then, the TIDE score, dysfunctional cytotoxic T cells, exclusion cytotoxic T cells, interferon gamma (IFNG) score and M2-TAMs, cancer-associated fibroblasts (CAFs), and myeloid-derived suppressor cell (MDSC) infiltrations were assessed by the TIDE platform to compare the antitumor and tumor immune escape abilities of the two MRGPI subgroups. TIDE is a computational framework that can use its built-in signatures and algorithm to calculate the degree of infiltration of dysfunctional T cells, M2-TAMs, CAFs, and MDSCs, as well as the IFNG score (reflecting cytotoxic killing of T cells). Then, the degree of infiltrating M2-TAMs, CAFs, and MDSCs in the TME was used to estimate the strength of the TME to exclude T cells, which was called the T-cell exclusion score. Finally, the TIDE score was obtained based on the T-cell dysfunction score and T-cell exclusion score to estimate the ability of the tumors to escape from immunity. Detailed principles and algorithms can be found in the articles published by Jiang et al. (42) and Fu et al. (43).

Finally, we referred to the gene sets published by He et al. reflecting many specific antitumor immunological functions (attached in **Supplementary Table S5**), 44) and used the ssGSEA algorithm to evaluate the immune and molecular characteristics between the different MRGPI subgroups.

In order to explore the prognostic value of the MRGPI for patients after immunotherapy, we respectively performed survival analysis in the IMvigor210 cohort and a Kidney renal clear cell carcinoma (KIRC) ICI cohort (45, 46). IMvigor210 cohort is a phase II clinical trial of atezolizumab (anti-PD-L1) therapy in patients with locally advanced or metastatic urothelial carcinoma. The primary follow-up information of this clinical trial is OS. The prognostic information and RNA-sequencing data of the IMvigor210 cohort were built into and can be publicly obtained by the IMvigor210CoreBiologies R package. The KIRC ICI cohort composes three prospective clinical trials of receiving nivolumab (anti-PD-1) therapy in clear cell renal cell carcinoma (ccRCC), including CheckMate 025, CheckMate 009, and CheckMate 010. The primary follow-up data of the study are progression-free survival (PFS) and OS in patients with ccRCC who received anti-PD-1 immunotherapy. The clinicopathological data, immunotherapy information, treatment response, prognostic information, and tumor sample RNA-sequencing data of this cohort were selflessly shared by Prof. Toni K. Choueir et al. (Department of Medical Oncology, Dana-Farber Cancer Institute, Harvard Medical School) at https://doi.org/10.1038/s41591-020-0839-y. The MRGPI scores of patients in the IMvigor210 cohort and KIRC ICI cohort were calculated according to the following formula: (MRGPI) =, where represents the regression coefficient between the gene (i) and patient prognosis and represents the expression level of the gene (i).

In addition, time-dependent receiver operating characteristic curve (ROC) curve analyses were used to obtain the area under curve (AUC) of various prognostic models, which can reflect the model’s ability of prediction. The AUC of the MRGPI was compared with the AUC of two immune-related scores, TIDE and TIS. TIDE is a score obtained by comprehensively assessing the ability of the TME to exclude T cells and the infiltration levels of dysfunctional T cells within the TME. It is often used to evaluate the immune evasion ability of tumors and the potential efficacy of immunotherapy and can be calculated online (http://tide.dfci.harvard.edu/) (43). The TIS is a set of 18 genes that are highly correlated with clinical response to ICIs, including IFN-gamma signaling pathway, T cells, and Natural killer (NK) cell-related genes. The TIS score is calculated as the average log2 scale-normalized expression of 18 characteristic genes (42).

### Drug Sensitivity Analysis and Potential Inhibitors Targeting Metabolism-Related Gene Prognostic Index Subgroups

We explored the relationship between gene expression and drug sensitivity using the Gene Set Cancer Analysis (GSCA) online tool (http://bioinfo.life.hust.edu.cn/GSCA/#/drug). The GSCA tools collected the IC50 of 367 small molecules in 987 cell lines from Genomics of Drug Sensitivity in Cancer (GDSC) and the IC50 of 481 small molecules in 860 cell lines from Cancer Therapeutics Response Portal (CTRP) (47–49). Gene expression sequencing data of these cell lines were simultaneously collected from the Cancer Cell Line Encyclopedia (CCLE) database. Afterward, the mRNA expression data and drug IC50 data were merged. Pearson correlation analysis was performed to get the correlation between gene mRNA expression and drug IC50. The p-values with adjusted FDR were obtained. A negative correlation means that gene expression is suppressed indicating sensitivity to that drug and *vice versa*.

The Connectivity map (cMAP) database (https://portals.broadinstitute.org/cmap/) is a public online tool that was used to identify candidate inhibitors for the MRGPI subgroups based on gene expression profiles. Differential expression genes between the high- and low-MRGPI groups were used to query in cMAP to get a list of potential inhibitors (50). All compounds’ mechanism of action (MoA) and drug targets were downloaded from clue (https://clue.io).

## Results

### Differentially Expressed Metabolism-Related Genes in Head and Neck Squamous Cell Carcinoma

Differential expression analysis was performed between HNSCC tumor samples (n = 502) and normal samples (n = 44), and finally, a total of 5,897 DEGs were obtained, including 4,473 upregulated genes and 1,424 downregulated genes (**Supplementary Figure S1A**). A total of 188 metabolism-related DEGs were then acquired by intersecting the lists of metabolism-related genes integrated from the KEGG database with the DEGs. Finally, 93 metabolism-related genes were found upregulated in tumor samples, while 95 genes were downregulated (**Supplementary Figure S1B**). The functional enrichment analysis showed that 188 metabolism-related DEGs were significantly associated with 690 GO terms and 54 KEGG pathways (details in **Supplementary Table S6)**, and the top 8 GO terms and KEGG pathways are shown in **Supplementary Figures S1C, D**.

### Metabolism-Related Hub Genes

To obtain the metabolism-related hub genes, WGCNA was carried out on the candidate genes (n = 188). The power value when the correlation coefficient between the connectivity K and the logarithm log(P(k)) reaches 0.85 is set as the β value. The optimal soft-thresholding power was 4 based on the scale-free network (**Supplementary Figure S2A**). Two modules were identified according to the average linkage hierarchical clustering and the optimal soft-thresholding power (**Supplementary Figures S2B, C**). According to the Pearson correlation coefficient between a module and sample feature for each module, the turquoise module is closely correlated with HNSCC tumors. Thus, the genes in the turquoise module were selected for further analysis. There were 23 genes and 99 edges of the turquoise module of the networks with a threshold weight >0.2 (**Supplementary Figure S2D**). The top 9 significantly enriched hallmark pathways for the genes in the turquoise module were shown in **Supplementary Figure S2E** (details in **Supplementary Table S7**). Among these 23 genes of the turquoise module, the expression of 17 metabolism-related hub genes was closely correlated with HNSCC patient OS as determined by Kaplan–Meier survival analysis, as shown in **Supplementary Figure S3** (p < 0.05, log-rank test).

### Survival Outcomes in the Different Metabolism-Related Gene Prognostic Index Groups

To further identify the metabolism genes that play as independent prognostic factors, multivariate Cox regression was carried out among the 17 metabolism-related hub genes. Finally, seven genes were recognized as independent factors, including HPRT1, AGPAT4, AMY2B, ACADL, CKM, PLA2G2D, and ADA. Therefore, the MRGPI of patients’ tumor samples was calculated by the formula: MRGPI = the expression level of HPRT1 * (0.57) + the expression level of AGPAT4 * (0.24) + the expression level of AMY2B * (-0.50) + the expression level of ACADL * (0.59) + the expression level of CKM * (0.07) + the expression level of PLA2G2D * (-0.21) + the expression level of ADA * (0.21).

**Figure 1:**
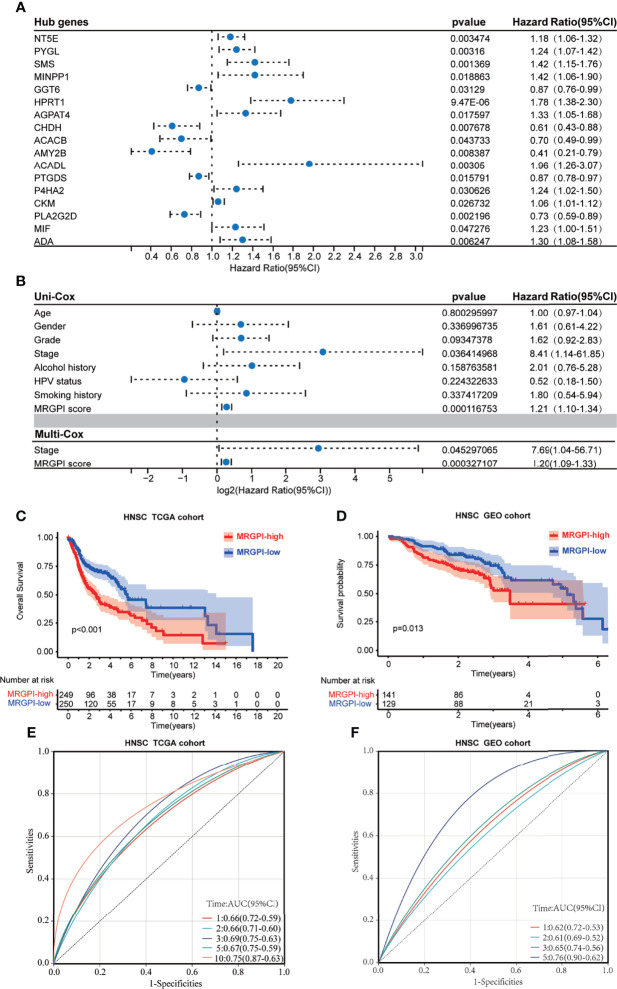
The prognostic role of the MRGPI. **(A)** Univariate Cox analysis of 17 metabolism-related hub genes. **(B)** Univariate Cox analysis of clinicopathologic factors and the MRGPI score and multivariate Cox analysis of the factors significant in the univariate Cox analysis (p < 0.05). **(C)** Kaplan–Meier analysis of the MRGPI subgroups in TCGA cohort. **(D)** Kaplan–Meier analysis of the MRGPI subgroups in the GEO cohort. **(E)** ROC analysis of the MRGPI on OS at 1-, 2-, 3-, 5-, and 10-year follow-up in TCGA cohort. **(F)** ROC analysis of the MRGPI on OS at 1-, 2-, 3-, and 5-year follow-up in the GEO cohort. MRGPI, metabolism-related gene prognostic index; TCGA, The Cancer Genome Atlas; GEO, Gene Expression Omnibus; ROC, Receiver operating characteristic curve.

The 499 HNSCC patients from TCGA were separated into the high-MRGPI group and low-MRGPI group according to the median MRGPI of all patients. Details of clinicopathological characteristics of the two MRGPI groups were exhibited in **Supplementary Table S8**, and the distribution of these characteristics has no difference between the two groups. Univariate Cox analysis indicated that Stage and MRGPI are significantly associated with the prognosis of HNSCC patients (**Figure 1A**). Further multivariate Cox regression analysis confirms that Stage and MRGPI are independent prognostic factors after being adjusted by other clinicopathologic factors (**Figure 1B**). Importantly, the OS of patients with low MRGPI was significantly higher than that of patients with high MRGPI (p < 0.001, log-rank test; **Figure 1C**). Similarly, the disease-specific survival (DSS) of patients with low MRGPI was also significantly better than that of patients with high MRGPI (p < 0.001, log-rank test; **Supplementary Figure S4A**). Moreover, according to the previous treatment of HNSCC patients in TCGA database, we divided the patients into radiation therapy subgroups, non-radiation therapy subgroups, molecular targeted therapy subgroups, and non-molecular targeted therapy subgroups. Excitingly, the MRGPI had an excellent ability to distinguish patient outcomes in all 4 subgroups, i.e., patients in the low-MRGPI group had better OS than those in the high-MRGPI group (p < 0.05, log-rank test; **Supplementary Figures S4C**–S4F).

In addition, we used an HNSCC dataset (GSE65858; n = 270) from the GEO database to externally validate the predictive effect of the MRGPI score on the prognosis. Coinciding with the results from TCGA cohort, low-MRGPI patients have better OS than that of high-MRGPI patients (p = 0.013, log-rank test; **Figure 1D**). Additionally, the patients with a low MRGPI have a longer disease-free interval (DFI) than that of high-MRGPI patients (p < 0.001, log-rank test; **Supplementary Figure S4B**). The model discriminatory accuracy was self-verified in TCGA training cohort using the AUC, resulting in values of 0.66 (95% CI: 0.59–0.72), 0.66 (95% CI: 0.60–0.71), 0.69 (95% CI: 0.63–0.75), 0.67 (95% CI: 0.59–0.75), and 0.75 (95% CI: 0.63–0.87) at 1, 2, 3, 5, and 10 years, respectively (**Figure 1E**), and verified by the GEO validation cohort, resulting in values of 0.62 (95% CI: 0.53–0.72), 0.61 (95% CI: 0.52–0.69), 0.65 (95% CI: 0.56–0.74), and 0.76 (95% CI: 0.62–0.90) at 1, 2, 3, and 5 years, respectively (**Figure 1F**), which both reflected satisfactory accuracy.

### Metabolic Characteristics of the Different Metabolism-Related Gene Prognostic Index Subgroups

**Figure 2:**
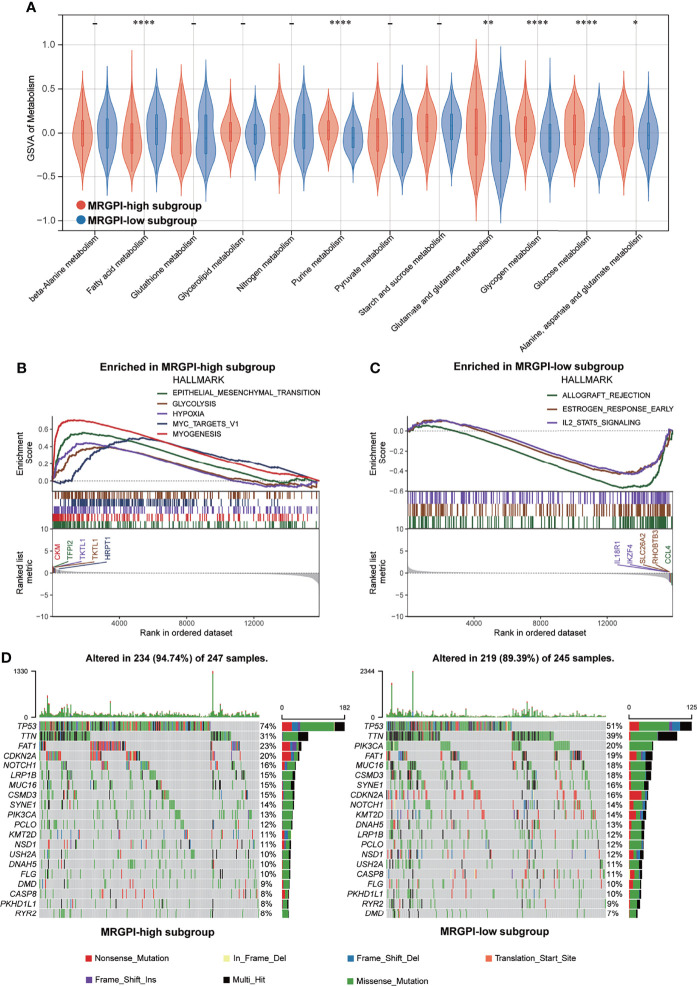
Metabolic activities and molecular characteristics of the different MRGPI subgroups. **(A)** The scores of common metabolic pathways of the different MRGPI subgroups. **(B)** Hallmark gene sets enriched in the high-MRGPI subgroup (p < 0.05, FDR <0.25). **(C)** Hallmark gene sets enriched in the low-MRGPI subgroup (p < 0.05, FDR <0.25). **(D)** Top 20 mutated genes in the different MRGPI subgroups. Mutated genes (rows) are ordered by mutation rate; samples (columns) are arranged to emphasize mutual exclusivity among mutations. The rightmost label shows mutation percentage, and the top shows the overall mutation number of the patients. The colored squares indicate the mutation type. *P < 0.05,**P < 0.01,****P < 0.0001. MRGPI, metabolism-related gene prognostic index; FDR, false discovery rate.

To gain further biological insight into the metabolic character of the MRGPI subgroups, we estimated the scores of common metabolic pathways. The metabolic scores indicated that the patients of the high-MRGPI group have higher levels of Purine metabolism; Glutamate and glutamine metabolism; Glycogen metabolism; Glucose metabolism; and Alanine, aspartate, and glutamate metabolism, while the patients in the low-MRGPI group have higher Fatty acid metabolism level (**Figure 2A**).

### Molecular Characteristics of the Different Metabolism-Related Gene Prognostic Index Subgroups

GSEA was performed to determine the specific gene sets enriched in each MRGPI subgroup. Against the background of gene sets included in the hallmark dataset, the gene set enriched in the high-MRGPI subgroup contains pathways related to tumor epithelial-mesenchymal transition (EMT) conversion, glycolysis, and hypoxia (**Figure 2B**), while the gene set of the low-MRGPI sample is enriched in interleukin-2 (IL-2) channels (**Figure 2C**; p < 0.05, FDR <0.25). When using the KEGG database as a background, the gene sets enriched in the high-MRGPI subgroup contains pathways related to PRIMARY_IMMUNODEFICIENCY, FOCAL ADHESION, and immune response (**Supplementary Figure S5A**), while the gene set of the low-MRGPI sample is rich in cancer and tumor metastasis-related pathways (**Supplementary Figure S5B**). The detailed results of GSEA are listed in **Supplementary Table S9**.

To further discover the different immunological natures brought about by metabolic characteristics, we explored the gene mutation landscapes of the different MRGPI subgroups. First, our results intuitively show that there are more samples with genetic mutations in the high-MRGPI subgroup than those in the low-MRGPI group (94.47% vs. 89.39%). Missense mutation accounted for the largest proportion (87.4%) of all mutation types, followed by Nonsense mutation (7.1%) and Frameshift deletions (2.9%) in the high-MRGPI subgroup. In the low-MRGPI group, the order of the proportions of the different mutation types is the same. Missense mutation is the most common mutation type (87.2%), followed by Nonsense mutation (7.0%) and Frameshift deletions (3.4%) in the high-MRGPI subgroup. Next, we explored the top 20 genes with the most frequent mutation rates in the two MRGPI subgroups (**Figure 2D**). Two MRGPI subgroups have obviously distinct mutant gene atlases. For example, although the gene with the highest mutation frequency in the two groups is TP53, the mutation rate of TP53 is as high as 74% in the high-MRGPI subgroup, which is much higher than the 51% mutation rate in the low-MRGPI group. Moreover, an important tumor suppressor gene, CDKN2A, which controls the cell cycle has an incidence rate of 20% in the high-MRGPI group and only 16% in the low-MRGPI group. Additionally, an important mutation that can cause the activation of the notch pathway, NOTCH1-mutation, has an incidence rate of 16% in the high-MRGPI group and only 14% in the low-MRGPI group.

### Relationship Between the Metabolism-Related Gene Prognostic Index Grouping and Seven-Step Anticancer Immune Response Processes

**Figure 3:**
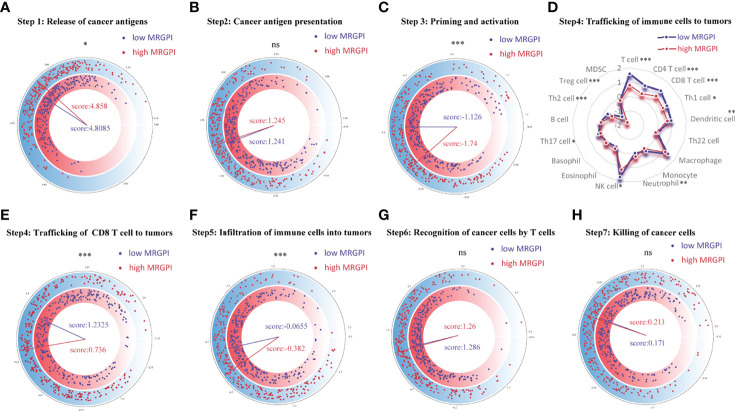
Correlation between scores of the seven-step cancer-immunity cycle and the MRGPI subgroups. **(A)** The quantity of release of cancer antigens (step 1 of the seven-step cancer-immunity cycle) between the MRGPI subgroups. **(B)** The quantity of cancer antigen presentation (step 2) between the MRGPI subgroups. **(C)** The ability of priming and activating immune cells (step 3) between the MRGPI subgroups. **(D)** The ability of trafficking kinds of immune cells to tumors (step 4) between the MRGPI subgroups. **(E)** The ability of trafficking CD8 T cells to tumors (step 4) between the MRGPI subgroups. **(F)** The degree of infiltration of immune cells into tumors (step 5). **(G)** The ability of recognizing cancer cells by T cells (step 6). **(H)** The ability of killing cancer cells (step 7) between the MRGPI subgroups. MRGPI, metabolism-related gene prognostic index.

The anticancer immune response can be conceptualized as a series of stepwise events, including the release of cancer antigens (step 1), cancer antigen presentation (step 2), initiation and activation (step 3), transportation of immune cells to the tumor (step 4), immune cells infiltrate the tumor (step 5), T cells recognize cancer cells (step 6) and kill cancer cells (step 7). The activity of these steps reflects the ability of the body’s immune cells to mobilize, transport, and kill, which together determine the success of the antitumor immune response. To analyze the impact of tumor metabolic activity on the seven-step anticancer immune response process of the 26 immune cells, we obtained signatures representing the seven-step anticancer immune process from the TIP database (attached in **Supplementary Table S2**) and used the ssGSEA algorithm to evaluate the ability of each antitumor step in patients. Then, we used the Wilcoxon test to compare the status of the seven antitumor immune processes in the different MRGPI subgroups (**Figure 3**). We found that the ability to release cancer antigens was stronger in the high-MRGPI group (**Figure 3A**). The ability of immune cell priming and activation of the low-MRGPI patients is significantly superior to that of the high-MRGPI patients (**Figure 3C**). The total ability of trafficking immune cells to tumors is stronger in the low-MRGPI group than in the high-MRGPI group. In detail, the trafficking abilities of T cell, CD4 T cell, CD8 T cell, Th1 cell, DC, NK cell, and Th2 cell to tumors are stronger in the low-MRGPI patients than those in the high-MRGPI patients, while the trafficking abilities of Neutrophil and Treg cell to the tumor are weaker in the low-MRGPI patients (**Figure 3D**). In the next step, the degree of immune cell infiltration into tumors is naturally higher in the low-MRGPI group (**Figure 3F**). However, the capabilities of cancer antigen presentation, recognition of cancer cells by T cells, and killing of cancer cells between the two MRGPI groups showed no difference (**Figures 3B–G, H** **)**.

### Immune Cell Infiltration and the Tumor Microenvironment Characteristics in the Different Metabolism-Related Gene Prognostic Index Subgroups

**Figure 4:**
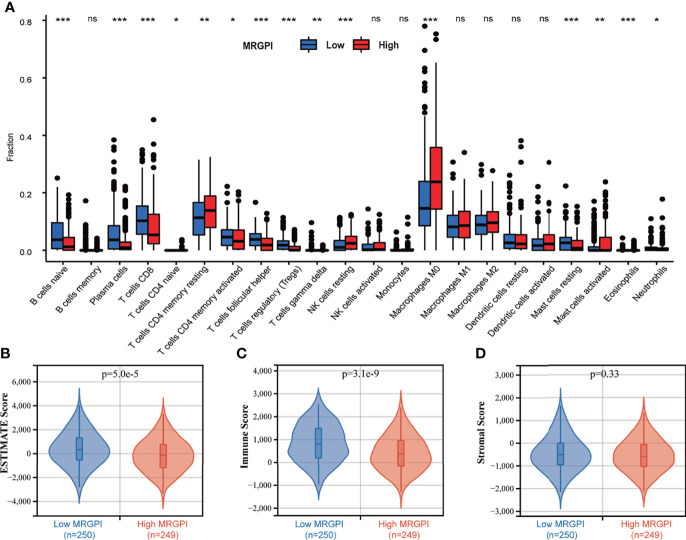
The TME landscape of the different MRGPI subgroups. **(A)** The proportions of immune cells within the TME in the different MRGPI subgroups. The scattered dots represent the immune score of the two subgroups. The thick lines represent the median value. The bottom and top of the boxes are the 25th and 75th percentiles (interquartile range), respectively. Significant statistical differences between the two subgroups were assessed using the Wilcoxon test (ns, not significant; *p < 0.05; **p < 0.01; ***p < 0.001). **(B)** The ESTIMATE Score (tumor purity) of the two MRGPI subgroups. **(C)** The Immune Score of the two MRGPI subgroups. **(D)** The Stromal Score of the two MRGPI subgroups. TME, tumor microenvironment; MRGPI, metabolism-related gene prognostic index.

To further assess the immune characteristics of the TME in the different MRGPI subgroups, we firstly used the CIBERSORT algorithm to evaluate the proportions of 22 immune cell infiltration of each HNSCC case in the different MRGPI subgroups (**Figure 4A**). We found that naive B cells, plasma cells, CD8 T cells, memory activated CD4 T cells, follicular helper T cells, Treg cells, gamma delta T cells, resting Mast cells, and Neutrophils were significantly more abundant in the low-MRGPI subgroup, while memory resting CD4 T cells, resting NK cells, M0 macrophages, and activated Mast cells showed a significantly high infiltration level in the high-MRGPI subgroup (**Figure 4A**).

Moreover, the ESTIMATE algorithm was used to evaluate the tumor microenvironmental components of each HNSCC sample. Finally, we calculated the ESTIMATE score (that infers tumor purity), immune score, and stromal score to compare the differences in the immune microenvironment between the low-MRGPI subgroup and the high-MRGPI subgroup. ESTIMATE score is the sum of the immune score and stromal score, which reflects the proportion of immune cells and stromal cells within the TME, and is also an indicator of tumor purity. A higher ESTIMATE score represents a higher proportion of immune cells and stromal cells within the TME and thus a lower proportion of tumor cells (lower tumor purity). That is, the ESTIMATE score is inversely proportional to tumor purity. We found that the low-MRGPI subgroup has a significantly higher ESTIMATE score (lower tumor purity) and immune score, which is consistent with the immune infiltration results above (p < 0.001) (**Figures 4B, C** **)**. There was no difference in stromal scores between the two subgroups (**Figure 4D**).

In addition, we further used certain gene signatures that can reflect specific antitumor immunological functions to evaluate the immune and molecular characteristics between the different MRGPI subgroups. The results showed that there is better antigen presentation ability [activated dendritic cells (aDCs), antigen presenting cells (APC)], more chemokine receptors (CCR), stronger anti-inflammatory and cytotoxic effects, and more IFN responses in the low-MRGPI group (**Supplementary Figure S6A**).

### The Benefit of Immune Checkpoint Inhibitor Therapy in the Different Metabolism-Related Gene Prognostic Index Subgroups

As the MRGPI can successfully distinguish the OS rate of HNSCC patients in different subgroups and is related to multiple steps in the antitumor immune response processes, we naturally guessed whether the MRGPI can predict the potential clinical efficacy of ICI therapy.

First, a correlation analysis was performed between the MRGPI and immune checkpoint proteins, including PD-1 (PDCD1), PD-L1 (CD274), CTLA-4, lymphocyte activation gene-3 (LAG-3), T-cell immunoglobulin, and immunoreceptor tyrosine-based inhibition motif domain (TIGIT) in the 502 HNSCC patients. There were significant negative correlations between the MRGPI and these immune checkpoint proteins (p < 0.01), except for PD-L1, which was not associated with the MRGPI (p > 0.05) (**Supplementary Figures S6B–F**). Specifically, a low MRGPI correlates with a high expression of checkpoint proteins, while a high MRGPI correlates with a low expression of checkpoint proteins. Many studies have demonstrated that a higher expression of immune checkpoint proteins such as PD-1 and CTLA-4 is associated with higher response rates or better efficacy of immunotherapy (51, 52). Therefore, these results suggest that patients with a low MRGPI may be more likely to benefit from immunotherapy.

**Figure 5:**
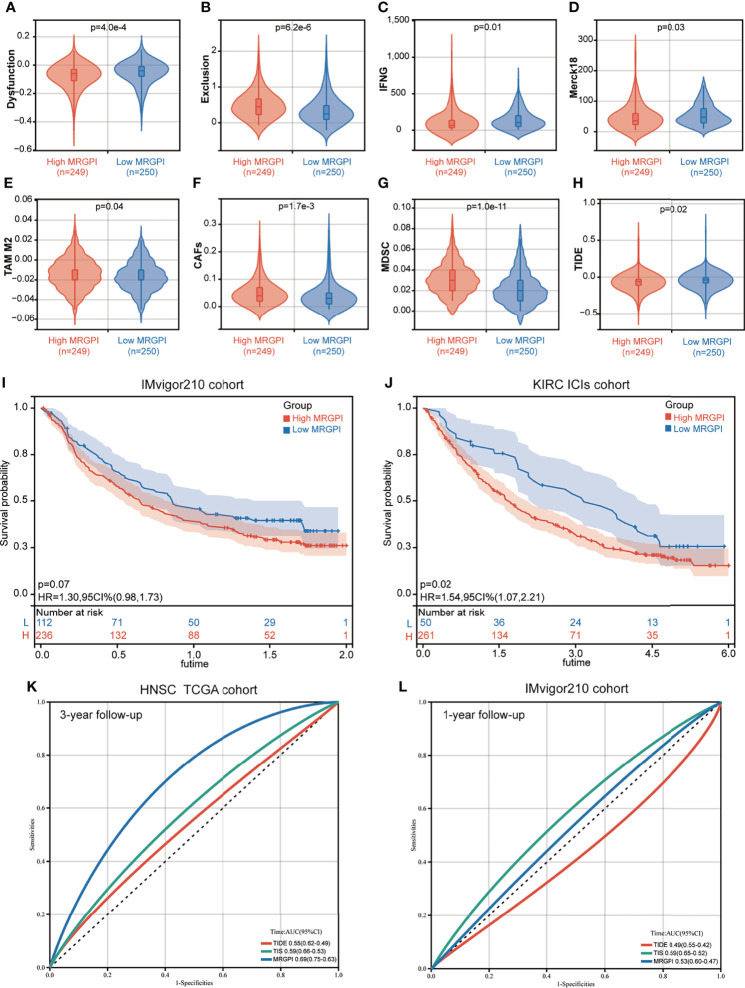
The prognostic significance of the MRGPI in patients with immune checkpoint inhibitor therapy. **(A–H)** T-cell dysfunction score, T-cell exclusion score, IFNG, Merck18, M2-TAMs, CAFs, MDSCs, and TIDE score in the different MRGPI subgroups. The scores between the two MRGPI subgroups were compared through the Wilcoxon test. **(I)** Kaplan–Meier analysis of the MRGPI subgroups in the IMvigor210 cohort. **(J)** Kaplan–Meier analysis of the MRGPI subgroups in the KIRC ICI cohort. **(K)** ROC analysis of the MRGPI, TIDE, and TIS on overall survival at 3-year follow-up in TCGA cohort. **(L)** ROC analysis of the MRGPI, TIDE, and TIS on overall survival at 1-year follow-up in IMvigor210.

We then used the TIDE algorithm to assess the potential clinical efficacy of immunotherapy in the different MRGPI subgroups. We found that the low-MRGPI patients with higher immune cell infiltration had higher T-cell dysfunction scores, while the high-MRGPI patients with less immune cell infiltration level had higher T-cell exclusion scores (**Figures 5A, B** **)**. Moreover, two signatures for assessing the ability to release IFN-γ, IFNG, and Merck18 both suggest that the patients of the low-MRGPI subgroup have a stronger IFN-γ release ability, which indirectly reflects that they have a stronger tumor-killing effect (**Figures 5C, D** **)**. In addition, three kinds of suppressor cells that contribute to tumor immune escape, M2-TAMs), CAFs, and MDSCs, respectively, suggest that the high-MRGPI tumors have a stronger immunosuppressive microenvironment and a more formidable immune evasion tendency (**Figures 5E–G**). Finally, the above results are comprehensively analyzed to get the TIDE prediction score of each HNSCC patient. A higher TIDE prediction score reflected a higher potential for immune evasion, which suggested that the patients were less likely to benefit from ICI therapy. In our results, the TIDE scores of the low-MRGPI subgroup were surprisingly slightly higher than those of the high-MRGPI subgroup, implying that the low-MRGPI patients may not benefit from ICI therapy compared with the high-MRGPI patients (**Figure 5H**).

As the result of the ICI therapy benefit predicted by TIDE is inconsistent with the result of the antitumor immune response in the MRGPI subgroups, we further estimated the prognostic value of the MRGPI in two cancer cohorts that had received anti-PD-L1 therapy. In the IMvigor210 cohort of bladder cancer, patients with a low MRGPI have better immunotherapy efficacy and better OS prognosis (**Figure 5I**). In the renal clear cell carcinoma (KIRC) cohort treated with nivolumab anti-PD-1 monoclonal antibody, we found that patients with a low MRGPI have significantly better ICI therapy efficacy and higher OS rates than patients with a high MRGPI (**Figure 5J**). To validate the performance of the MRGPI on patient prognosis, we compared the performance of the MRGPI with TIDE and TIS in TCGA HNSCC cohort and IMvigor210 cohort. The AUC of the MRGPI was better than the AUC of TIS and TIDE at 3 years’ follow-up in TCGA cohort that included patients with comprehensive therapy (**Figure 5K**). However, in the cohort receiving ICI therapy, the AUC of the MRGPI was between TIS and TIDE at 1-year follow-up (**Figure 5L**). Hence, we considered that the MRGPI is an ideal predictive index whose predictive power for OS is better than TIDE in the ICI therapy cohort.

### Drug Sensitivity Analysis and Potential Inhibitors Targeting Metabolism-Related Gene Prognostic Index Subgroups

**Figure 6:**
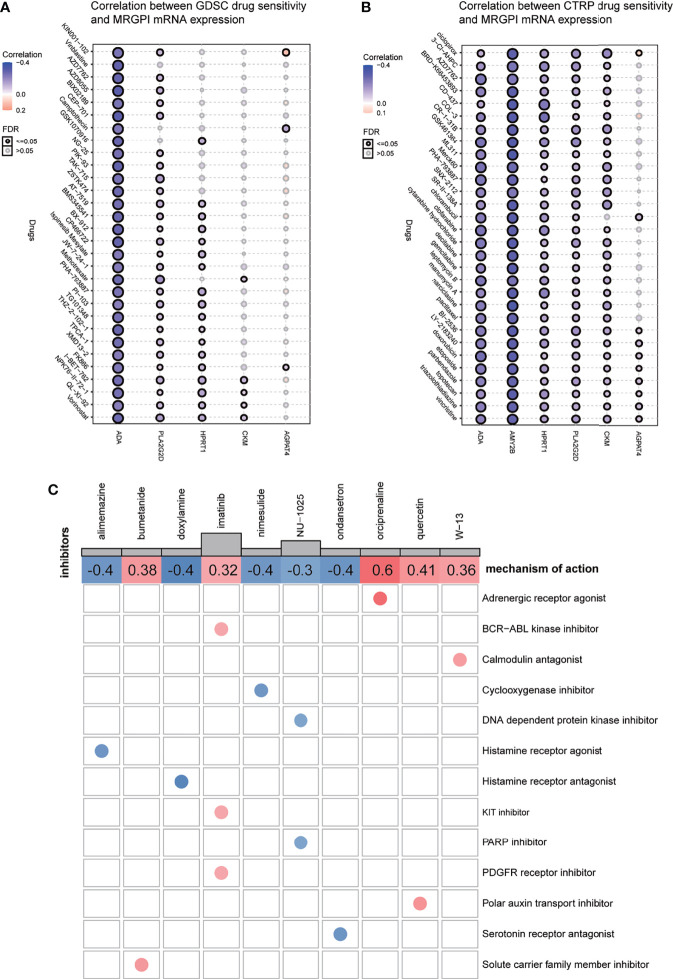
Drug sensitivity analysis and potential inhibitors targeting the MRGPI subgroups. **(A)** Correlation between GDSC drug sensitivity and the MRGPI gene expression. **(B)** Correlation between CTRP drug sensitivity and the MRGPI gene expression. **(C)** cMAP analysis in the different MRGPI subgroups. MRGPI, metabolism-related gene prognostic index; GDSC, Genomics of Drug Sensitivity in Cancer; CTRP, Cancer Therapeutics Response Portal; cMAP, Connectivity map.

IC50, the 50% inhibitory concentration, represents the concentration of a drug that is required for 50% inhibition of tumor cells. It is commonly used as a measure of drug sensitivity. That is, the lower the IC50 value of a drug on tumor cells, the more sensitive the tumor cells are to this drug. For drug sensitivity analysis using the GDSC database, the IC50 values for the treatment of 987 tumor cells by 367 drugs, respectively, and transcriptome sequencing data for these tumor cell lines [based on RNA microarrays and normalized using a robust multi-array analysis (RMA) algorithm] were first obtained from the GDSC database. Then, the expression values of the candidate genes in each cell line were combined with the IC50 values of each drug for each cell line using the cell line name as a reference. Finally, correlation analysis was performed on gene expression values and drug IC50 values using Pearson correlation, and then, the correlation between candidate genes and each drug was calculated. For drug sensitivity analysis using the CTRP database, the IC50 values for the treatment of 860 tumor cells by 481 drugs were obtained from the CTRP database, and transcriptome sequencing data for these tumor cell lines [based on RNA microarrays and normalized, quantified by the RNA-Seq by Expectation Maximization (RSEM) algorithm] were obtained from the CCLE database. Then, the candidate gene expression levels and drug IC50 values were combined, and their correlations were calculated according to the above method. Because the drugs and tumor cells tested in the GDSC database and the CTRP database are different, the results obtained are also different. For ACADL, only one drug in both databases has a meaningful correlation of IC50 with its expression and is therefore not exhibited in **Figures 6A, B**. The AMY2B gene is not embodied in the GDSC database, so the correlation between AMY2B and drug sensitivity is not shown in **Figure 6A**. The drugs that sensitized the rest of the MRGPI genes were ranked by the integrated level of correlation coefficient and FDR (adjust. p-value). The top 30 ranked drugs were shown in **Figures 6A, B**. In the figure, the purple dots represent a negative correlation between the gene and the IC50 of the candidate drug. This means that the gene is positively correlated with the sensitivity of the candidate drug. We found that the ADA expression was positively correlated with sensitivity to classical chemotherapeutic agents, such vinblastine, camptothecin, gemcitabine, and methotrexate, as well as novel small-molecule inhibitors, such as AT-7519 [Cyclin-dependent kinases (CDK) inhibitor], AZD7762 [Checkpoint Kinase (CHK) inhibitor], and AZD8055 [mammalian target of rapamycin (mTOR) inhibitor]. PLA2G2D was negatively associated with the resistance to vincristine, methotrexate, GSK461364 (PLK1 inhibitor), parbendazole (microtubule inhibitor), and so on. HPRT1 was significantly positively correlated with sensitivity to CD-437 [Retinoic Acid Receptor gamma (RARγ) agonist], COL-3 [Matrix metalloproteinase (MMP) inhibitor], and manumycin A (antibiotic). CKM was significantly positively correlated with sensitivity to ciclopirox (antifungal agent), CR-1-31B (eIF4A inhibitor), and narciclasine. AGPAT4 was significantly positively correlated with resistance to KIN001-102 and ciclopirox and negatively correlated with resistance to camptothecin, chlorambucil, and triazolothiadiazine.

Potential compounds targeting the MRGPI subgroups were identified with the DEGs between the high- and low-risk groups by querying the cMAP database. The potential compounds specific to the MRGPI and the corresponding mechanism of action are shown in **Figure 6C**. The negative mean value indicated that changes in expression profiles of these drug-treated cell lines in the cMAP database were reversed from those in the high-risk group and the positive value was just the opposite. Therefore, alimemazine, doxylamine, nimesulide, NU-1025, and ondansetron may serve as potential inhibitors targeting the MRGPI in the high-risk group.

## Discussion

Although immunotherapy is of great benefit to the survival of patients with relapsed and refractory HNSCC, the low response rate to treatment limits the clinical application of ICIs. Moreover, increasing evidence has shown that metabolic reprogramming in the TME can affect the efficacy of immunotherapy by weakening the antitumor immunity of immune cells. Therefore, it is particularly important to construct a metabolism-related prognostic biomarker to identify the patient groups that are likely to benefit from conventional therapies and ICI therapy and profile their tumor molecular characteristics and TME landscapes.

In our research, we used the WGCNA algorithm to screen 17 metabolism-related hub genes that are most related to tumorigenesis and affect the patient’s survival. Among these hub genes, only genes that were independent prognostic factors for OS were used to develop the MRGPI, including HPRT1, AGPAT4, AMY2B, ACADL, CKM, PLA2G2D, and ADA. In two independent cohorts of TCGA and GEO database, both survival analysis and AUC proved that the MRGPI is an effective prognostic indicator of HNSCC, with better survival in the high-MRGPI patients and worse survival in the low-MRGPI patients.

The MRGPI is composed of seven genes: HPRT1, AGPAT4, AMY2B, ACADL, CKM, PLA2G2D, and ADA. HPRT1 is an important enzyme involved in purine metabolism, which is often used as a housekeeping gene. However, increasing evidence has unraveled the mystery of HPRT1 as a potential biomarker in a variety of tumors. It has been reported that HPRT1 is significantly elevated in multiple cancer types (53, 54) and is associated with a poor prognosis due to the increased demand for nucleotide synthesis during tumor cell proliferation (55, 56). In both HNSCC and its subclass oral squamous cell carcinoma, multiple studies have demonstrated that HPRT1 was overexpressed and significantly correlated with the poor prognosis of patients (57, 58). HPRT1 was also found to be associated with drug resistance, such as cisplatin, in cancer cells and has been proposed as a therapeutic target of chemotherapeutic drugs (58, 59). Moreover, it has been reported that HPRT expression was negatively correlated with the infiltration of several immune cell subsets, including CD8+ T cells, CD4+ T cells, B cells, macrophages, neutrophils, and DCs (60). Additionally, the upregulation of HPRT in malignant tissue leads to an immunosuppressive microenvironment by directly promoting purine synthesis and then reducing immune cell activation (60). AGPAT4, also known as lysophosphatidic acid acyltransferase δ, is an emerging protein found to be involved in lipid metabolism reprogramming. It has been reported that a high expression of AGPAT4 in tumor cells can reduce the release of lysophosphatidic acid (LPA) (61, 62). Then, the reduced LPA reprograms the lipid metabolism of macrophages and promotes the polarization of macrophages to M2 macrophages, thereby inhibiting T-cell activation and facilitating tumor growth (63). Acyl-CoA dehydrogenase long-chain (ACADL), also be called LCAD, is a key enzyme that regulates the metabolic pathway of mitochondrial fatty acid oxidation by catalyzing the β-oxidation of long-chain fatty acids (64, 65). It has been reported that the expression of ACADL protein was positively associated with malignant progression in prostate cancer (66). However, Hill et al. (67) revealed that ACADL methylation is increased in breast cancer tissues and is associated with poor survival of breast cancer patients, which suggested that ACADL may play a tumor-suppressor role in breast cancer. Moreover, in hepatocellular carcinoma (HCC), the expression of ACADL was found significantly decreased in tumor tissues compared to normal liver tissues in both mRNA and protein levels, and restored ACADL expression suppressed HCC cell growth (68). Furthermore, it has been reported that the loss of ACADL enhanced tumor growth by inhibiting the expression of the suppressor gene phosphatase and tensin homolog deleted on chromosome ten PTEN) *in vivo*; therefore, the loss of ACADL is correlated with poor clinical prognosis of HCC patients (69). The mechanism behind ACADL inhibiting PTEN is perhaps due to the loss of ACADL leading to accumulation of unsaturated fatty acids. Then, unsaturated fatty acids inhibit PTEN *via* miR-21 upregulation in hepatocytes (70). Creatine kinase (CK), also known as creatine phosphokinase or phosphocreatine kinase, is an extremely important enzyme catalyzing the conversion of adenosine diphosphate (ADP) to adenosine triphosphate (ATP), which plays a central role in energy homeostasis in the tissues displaying high and variable rates of energy turnover such as cardiac, brain, muscle, skeletal, and retina (69, 71). It has been known for about 40 years that aberrant CK levels are associated with various cancers (72). A review summarized that CK can regulate cell cycle progression by affecting the intracellular energy status and by influencing signaling pathways that are essential to activate cell division and cytoskeleton reorganization. Therefore, the involvement of CK in cell cycle regulation and cellular energy metabolism makes it a potential diagnostic biomarker and therapeutic target in cancer (72). Another study revealed that CK had a close relationship with bone and lymph node metastases and could be used as independent factors to predict a poor prognosis in lung cancer patients (72, 73). Recently, a study indicated that CKB plays an unexpected role in modulating T cell receptor (TCR)-mediated signaling and critically regulates the activation, proliferation, and cytokine secretion of T cells (74). However, there is still a lack of research on the role of CKM in cancer biology. PLA2G2D (sPLA2-IID) is a member of the phospholipase A2 family that hydrolyzes the sn-2 fatty acid ester bond of glycerophospholipids to produce lysophospholipids and free fatty acid (75). Miki et al. (76) found that compared with normal mice, Pla2g2d deficiency (Pla2g2d−/−) mice had a significantly reduced incidence of 7,12-Dimethylbenz(a)anthracene/12-O-tetradecanoylphorbol-13-acetate (DMBA/TPA)-induced skin carcinogenesis. However, the expression of PLA2G2D has also been found to be positively correlated with better prognosis in human HNSCC and breast cancer, which is also consistent with the result of our survival analysis (77, 78). Interestingly, PLA2G2D was found serving as a vital regulator of respiratory dendritic cell (rDC) activation and enhanced priming of virus-specific T cells after infections with respiratory viruses (79). ADA is a key enzyme in purine metabolism, which maintains the balance of adenosine inside and outside the cell by catalyzing the irreversible deamination of adenosine and converting adenosine into inosine (80). Several previous studies have shown that altered ADA activity is related to the progression of a variety of tumors, especially in breast cancer (81–84). A recent study revealed the changes in ADA2 activity that may contribute to the differentiation of macrophages into an unfavorable protumor M2 phenotype in triple-negative breast cancer (84).Together, these findings indicated that the genes that make up the MRGPI are involved in several metabolic activities, and most of the genes are related to tumor progression and prognosis. Therefore, this can explain to a certain extent why the MRGPI can be used as an independent prognostic factor for HNSCC and can predict the survival of patients. More interestingly, the abovementioned studies have shown that many genes of the MRGPI are involved in the metabolic reprogramming of immune cells, which in turn regulates the differentiation and activation of immune cells. Therefore, these results suggest that the MRGPI may be a potential indicator that can simultaneously reflect the metabolic activity and immune status in the TME.

To gain further biological insight into the metabolic character of the MRGPI subgroups, we then estimated the scores of common metabolic pathways. The metabolic scores of the different MRGPI subgroups indicated that the patients of the high-MRGPI group have higher levels of Purine metabolism; Glutamate and glutamine metabolism; Glycogen metabolism; Glucose metabolism; and Alanine, aspartate, and glutamate metabolism, while the patients in the low-MRGPI group have higher Fatty acid metabolism level. Purines are the most elementary metabolic substrates of all organisms, providing essential components for DNA and RNA syntheses. In addition, purines also provide the necessary energy and cofactors for maintaining cell survival and proliferation. Therefore, purines and their derivatives are widely involved in tumor progression, as well as immune responses and host–tumor interactions (85, 86). A recent study suggested that blocking the purine synthesis pathway significantly inhibited the tumorigenesis and stem-like properties of lung cancer cells (87). Previous studies have shown that cancer cells exhibit increased consumption and dependence on glutamine. The enhanced glutamine in cancer cells activates mTOR signal transduction, inhibits endoplasmic reticulum stress, and promotes protein synthesis, thereby promoting tumor growth and proliferation (88–90). Moreover, our previous research revealed that glutamine metabolism regulators were associated with poorer cancer prognoses and an immunosuppressive TME (91). In the TME under hypoxic conditions, Glucose metabolism is the most common metabolic reprogramming of tumors that plays a major role in cancer survival, proliferation, metastasis, and treatment resistance (92–96). Fatty acids are also an important class of metabolites, which are required for the synthesis of tumor cell membranes and signaling molecules in cellular proliferation (97). In our current study, the high-MRGPI patients, with a worse prognosis, have higher levels of Purine metabolism, Glutamate and glutamine metabolism, Glycogen metabolism, and Glucose metabolism, which are more conducive to tumor progression. This answers from another perspective why the MRGPI is related to the prognosis of HNSCC patients.

To further comprehend the immunological properties of the MRGPI subgroups, we explored the gene mutation profile of the different MRGPI subgroups. There are huge mutation differences between the patients of the high-MRGPI group and the low-MRGPI group. The most obvious difference is that the high-MRGPI patients have more TP53 mutation frequencies (74%) than the low-MRGPI patients (51%). TP53 is a well-known tumor suppressor gene, which exerts a tumor suppressor effect by controlling cell proliferation and promoting cell apoptosis (98–101). It has been reported TP53 mutations are positively correlated to shorter survival time and therapeutic resistance to radiotherapy and chemotherapy in HNSCC patients (102). Therefore, the worse prognosis of patients in the high-MRGPI group may be related to the higher frequency of TP53 mutations.

As most of the genes that make up the MRGPI participate in the processes of immune cell differentiation, activation, and antitumor immunity, it inspired us to further explore the relationship between the MRGPI and the antitumor immunity process, immune cell infiltration, and the condition of the tumor immune microenvironment. In the seven-step anticancer immune response processes, our evaluated results indicated that the high-MRGPI group has a stronger ability to release cancer antigens, which may be caused by more gene mutations in the high-MRGPI group. Interestingly, the high-MRGPI group has a higher ability to release antigens, as one would expect that this could be a positive feature for antitumor response and response to therapy. However, the ability of immune cell priming and activation, the ability of trafficking immune cells to tumors, and the degree of immune cell infiltration within tumors of the low-MRGPI subgroup were significantly superior to those of the high-MRGPI subgroup. In particular, the immune cells that play major roles in killing tumors, such as CD8 T cells, CD4 T cells, and NK cells, have stronger activation and driving in patients with a low MRGPI. In summary, these findings indicate that patients with a low MRGPI have a stronger activation and trafficking of immune cells and a higher abundance of immune cell infiltration, which leads to a stronger antitumor response and consequently a better prognosis for patients. The immune cell differences between the different MRGPI groups were then predicted in more detail. The results revealed that immune cells with antitumor effects such as plasma cells, CD8 T cells, memory-activated CD4 T cells, follicular helper T cells, and gamma delta T cells are significantly more abundant in the low-MRGPI subgroup. What is more interesting is that the cells that are considered to have no antitumor effect such as memory resting CD4 T cells, resting NK cells, and M0 macrophages were higher infiltrated in the high-MRGPI subgroup. Moreover, the TME Estimate scores indicated that the patients in the low-MRGPI group have higher immune scores than those in the high-MRGPI patients, while the stromal scores have no difference between the two groups. These results once again proved that the patients in the low-MRGPI group have more abundant antitumor immune cells within tumors and therefore have a better prognosis. Altogether, the evidence above suggested that the high-MRGPI group has a “desert-like” immune environment, with fewer anticancer immune cells and more cancer-promoting immune cell infiltration, and has weaker anticancer immune activity in multiple steps of the antitumor immune processes. These explain why the MRGPI is related to the prognosis of HNSCC patients from the perspective of antitumor immunology.

Considering the excellent prognostic effect of the MRGPI on traditional treatment and the close relationship between the MRGPI and antitumor immune response, we used the TIDE algorithm to predict the prognostic effect of the MRGPI on ICI therapy. The TIDE algorithm reflects the benefits of ICI treatment by estimating the potential of tumor immune escape. TIDE algorithm evaluated the tumor immune evasion ability from two perspectives: T-cell dysfunction score and T-cell exclusion score. T-cell dysfunction is a method to assess tumor immune escape by estimating the level of dysfunctional T cells infiltrating within the TME. T-cell exclusion is an assessment of the ability of the TME to prevent T-cell infiltration by the infiltration level of immunosuppressive cells including CAFs, MDSCs, and M2-TAMs in the TME. There was no direct connection between them, but tumor samples with a high T-cell infiltration generally had higher T-cell dysfunction and lower T-cell exclusion. In our study, the high-MRGPI patients had less cytotoxic T lymphocyte (CTL) infiltration and higher T-cell exclusion score (but not T-cell dysfunction score), M2-TAMs, CAFs, and MDSCs than those of the low-MRGPI patients, so their lower ICI response might be due to immune evasion *via* T-cell exclusion and more protumor immune cell infiltration (42, 103–105). On the contrary, the low-MRGPI group had a higher T-cell dysfunction score, which is associated with the more abundant CTL infiltration in their TME. Notably, there were subtle differences in the infiltration levels of M2 macrophages/M2-TAMs between the two MRGPI subgroups shown in **Figures 4B**, **5E.** This is because the infiltration level of M2 cells in **Figure 4B** was assessed according to the algorithm of the CIBERSORT software. CIBERSORT uses the deconvolution method to first extract the characteristics of various immune cells from single-cell RNA-seq and then reversely calculate the proportion of various immune cell components in tumor Bulk-seq, while the degree of infiltration of M2-TAMs in **Figure 5E** was estimated according to the TIDE algorithm. In addition, the two algorithms have different definitions of M2 macrophages/TAMs. Therefore, there will be subtle differences in the levels of assessed M2 macrophage infiltration. Interestingly, despite the differences between the aforementioned CIBERSORT and TIDE algorithms, their results were potentially relevant. As shown in **Figure 4B**, the low-MRGPI group has a higher CD8 T-cell infiltration assessed using the CIBERSORT algorithm, and thus it has a higher T-cell dysfunction score (**Figure 5A**) and a lower T-cell exclusion score according to the TIDE algorithm (**Figure 5B**). To our surprise, the low-MRGPI group had a higher TIDE score than the high-MRGPI group, which suggested that the tumors of these patients are more possible to immune escape (106). This result clearly contradicts the better survival of patients with a low MRGPI. Since the TIDE score calculated by the TIDE algorithm is based on the T-cell dysfunction score in tumor samples with high infiltrating CTLs and the T-cell exclusion score in samples with low infiltrating CTLs. In our study, most of the HNSCC samples have high infiltration of CTLs; thus, the TIDE score was mainly determined by the T-cell dysfunction score. Compared with patients in the high-MRGPI group, the low-MRGPI group had more T cells infiltrating the dysfunction, thus resulting in a higher TIDE score. Therefore, we speculate that the TIDE algorithm tends to use the infiltration degree of CTLs (mainly dysfunctional CTLs in our study) to calculate the degree of benefit of patients from immunotherapy, without comprehensively considering the role of other immune cells in antitumor, which leads to the inconsistent prediction of TIDE with survival analysis.

Therefore, to further confirm the prognostic value of the MRGPI, we performed survival analysis in two cancer cohorts that had received ICI therapy. The results of both clinical trials demonstrated that patients in the low-MRGPI group are more likely to benefit from immunotherapy and have a better OS rate than those in the high-MRGPI group. These results from the real world, rather than the prediction *in silico*, are consistent with our evidence for the role of the MRGPI in predicting immunotherapy efficacy. Several biomarkers have been proven to have good performance in predicting the efficacy of immunotherapy, such as TIDE and TIS. The TIDE score has been shown to be more accurate than PD-L1 levels and TMB in predicting the therapeutic outcome of malignant melanoma patients treated with anti-PD-1 or anti-CTLA-4 antibodies (42). TIS is a signature composed of 18 T cell-related genes, of which good predicted efficacy has been validated in two HNSCC cohorts treated with pembrolizumab and exhibited a positive correlation with treatment response and better survival (107). Therefore, we compared the ability of the MRGPI with TIDE and TIS to predict prognosis in the cohort receiving comprehensive therapy and immunotherapy. The AUC of the MRGPI was better than the AUC of TIS and TIDE at 3 years’ follow-up in TCGA cohort that included patients with comprehensive therapy. In the cohort receiving ICI therapy, the AUC of the MRGPI was between TIS and TIDE at 1-year follow-up. Hence, we considered that the MRGPI is an ideal predictive index whose predictive power for OS is comparable to TIDE and TIS in the ICI therapy cohort.

Finally, for the individualized precision therapy to patients with a high death risk or the high-MRGPI patients, we explored the potential compounds targeting the MRGPI genes and the potential existing small-molecule drugs targeting the MRGPI subgroups. For example, when faced with an HNSCC patient who failed multi-line therapies and the gene sequencing results showed that the key enzyme of purine metabolism-ADA is highly expressed, clinicians can try to use novel small-molecule inhibitors, such as AT-7519 (CDK inhibitor), AZD7762 (CHK inhibitor), and AZD8055 (mTOR inhibitor) for treatment. If it is more coincidental that the mTOR pathway in the patient’s cancer tissue is activated, then mTOR inhibitor AZD8055 can be more accurately selected for treatment. On the other hand, when the patient’s high-MRGPI score is calculated based on the gene sequencing results, drugs that target the MRGPI upregulated genes can be selected, such as alimemazine (histamine receptor agonist), doxylamine (histamine receptor antagonist), nimesulide (cyclooxygenase inhibitor), NU-1025 [DNA-dependent protein kinase inhibitor/Poly ADP-Ribose Polymerase (PARP) inhibitor], and ondansetron (serotonin receptor antagonist) for adjuvant therapy.

There are still many limitations in our study. Topping the list, there is a lack of a self-built HNSCC patient cohort to validate the predictive performance of the MRGPI. It is not optimal to use the GEO cohort to validate predictive models built by TCGA cohort, as their sequencing data are generated from different platforms and sequencing technologies. Second, our study requires more original laboratory findings or clinical observation to verify the role of the genes that constitute the MRGPI. Finally, although the MRGPI has acceptable predictive power (AUC) for long-term survival in HNSCC patients, its predictive power for short-term survival is not satisfactory.

**Figure 7:**
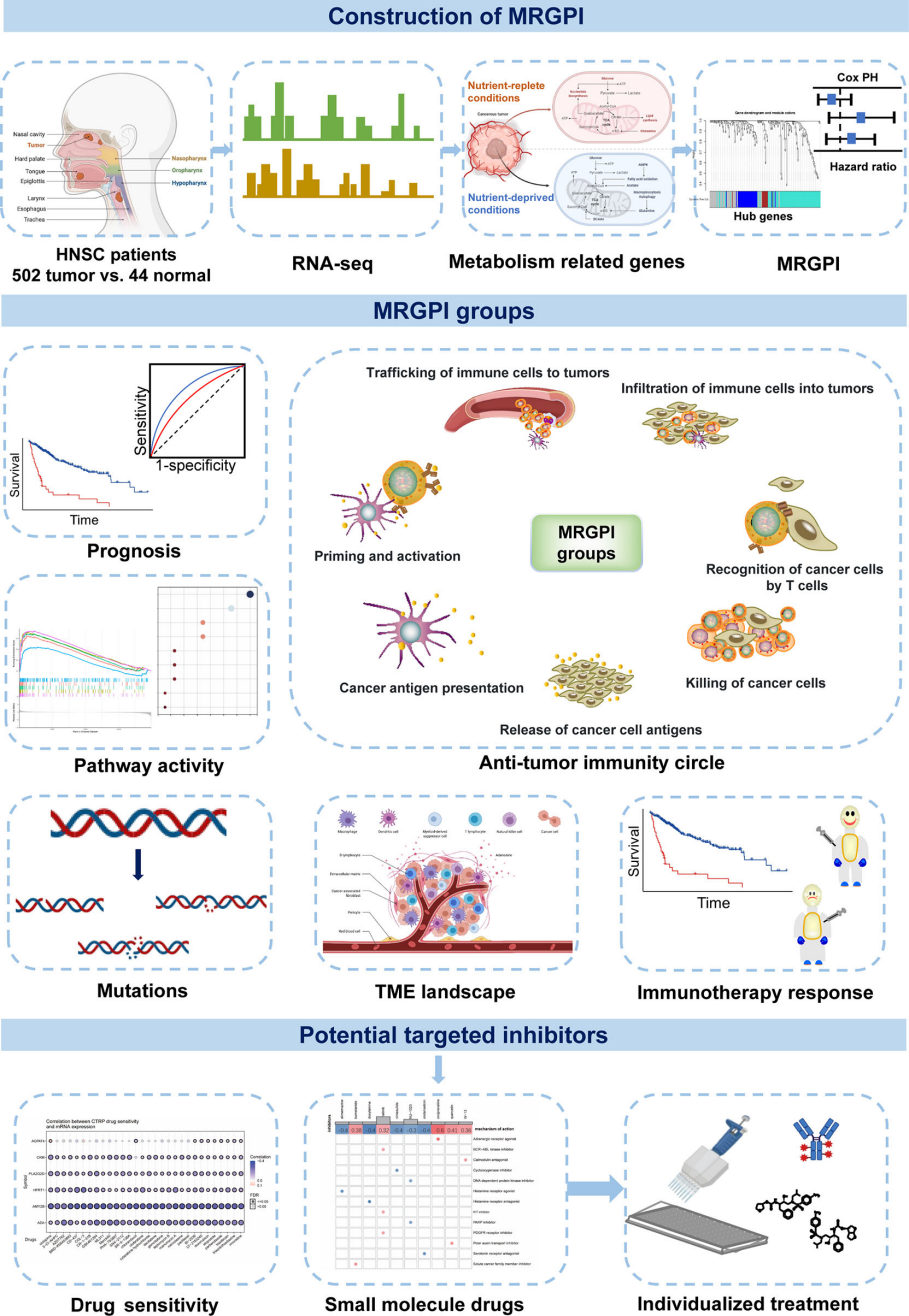
Graphical abstract for comprehensive characterization of the MRGPI subgroups in HNSCC.

In summary, the MRGPI is a promising metabolism-related prognostic biomarker. The MRGPI grouping may help in distinguishing metabolic and immune characteristics and predicting patient outcomes. The MRGPI also may serve as a potential prognostic biomarker for ICI immunotherapy, but further research is needed to confirm its efficacy (**Figure 7**).

## Data Availability Statement

The datasets presented in this study can be found in online repositories. The names of the repository/repositories and accession number(s) can be found in the article/**Supplementary Material**.

## Author Contributions

KD and JZ designed the project. BW, XH, and CL performed data extraction and analysis. TX, MK, and PS performed the quality assessment, and KD contributed to the article drafting. YY and YT revised the article critically and supervised the project. All authors read and approved the final article.

## Funding

This research was supported by The National Natural Science Foundation of China (Grant No. 81773354) and The Science and Technology Program of Guangzhou, China (Grant No. 202102020034).

## Conflict of Interest

The authors declare that the research was conducted in the absence of any commercial or financial relationships that could be construed as a potential conflict of interest.

## Publisher’s Note

All claims expressed in this article are solely those of the authors and do not necessarily represent those of their affiliated organizations, or those of the publisher, the editors and the reviewers. Any product that may be evaluated in this article, or claim that may be made by its manufacturer, is not guaranteed or endorsed by the publisher.

## Supplementary Material

Supplementary Figure 1 Differentially expressed metabolism-related genes in HNSCC. **(A)** Volcano map exhibiting all differentially expressed genes (DEGs) between 502 HNSCC samples and 44 para-cancer samples (p < 0.05, |log2FC| > 0.585). **(B)** Volcano map displaying metabolism-related DEGs between 502 HNSCCC samples and 44 para-cancer samples. **(C)** Gene Ontology (GO) enrichment analysis of the metabolism-related DEGs (p < 0.05). **(D)** Kyoto Encyclopedia of Genes and Genomes (KEGG) pathway analysis of the metabolism-related DEGs (p < 0.05).

Supplementary Figure 2 Identification of metabolism-related hub genes by the WGCNA. **(A)** The soft-thresholding power was 0.85 determined by the red horizontal line in the Weighted gene coexpression network analysis (WGCNA) analysis and the optimal soft threshold for WGCNA was 4. **(B)** WGCNA of metabolism-related differentially expressed genes with a soft threshold β = 4. **(C)** Gene modules related to HNSCC obtained by WGCNA. **(D)** The network of the genes in the turquoise module (weight of edge > 0.2). **(E)** Hallmark enriched in the genes of the turquoise module (p < 0.05).

Supplementary Figure 3 Kaplan-Meier curves of 17 metabolism-related hub genes. Kaplan-Meier survival analysis of 17 metabolism-related genes in TCGA cohort.

Supplementary Figure 4 The prognostic role of MRGPI. **(A)** Kaplan-Meier analysis of MRGPI subgroups on disease-free survival (DFS) in TCGA cohort. **(B)**. Kaplan-Meier analysis of MRGPI subgroups on disease-free survival (DFS) in the GEO cohort. **(C)** Kaplan-Meier analysis of MRGPI subgroups for radiotherapy patients. **(D)** Kaplan-Meier analysis of MRGPI subgroups for non-radiotherapy patients. **(E)** Kaplan-Meier analysis of MRGPI subgroups for molecular targeted therapy patients. **(F)** Kaplan-Meier analysis of MRGPI subgroups for non-molecular targeted therapy patients.

Supplementary Figure 5 Gene sets enriched in different MRGPI subgroups **(A)** KEGG gene sets enriched in MRGPI-high subgroup (P < 0.05, FDR < 0.25). **(B)** KEGG gene sets enriched in MRGPI-low subgroup (P < 0.05, FDR < 0.25).

Supplementary Figure 6 The molecular and immune features of MRGPI. **(A)**. The molecular and immune-related function of the different MRGPI subgroups. The gene sets of molecular and immune-related function were analyzed by the single sample gene set enrichment analysis (ssGSEA) and then compared between different MRGPI subgroups. The scattered dots represent the ssGSEA scores of the two subgroups. The thick lines represent the median value. The bottom and top of the boxes are the 25th and 75th percentiles (interquartile range), respectively. Significant statistical differences between the two subgroups were assessed using the Wilcoxon test. The relationship between MRGPI and immune checkpoint genes, such as **(B)** PD-L1, **(C)** PD-1, **(D)** CTLA-4, **(E)** LAG3, and **(F)** TIGIT.
