# Supplementary figures and images for: A Metabolism-Related Gene Prognostic Index Bridging Metabolic Signatures and Antitumor Immune Cycling in Head and Neck Squamous Cell Carcinoma
Source: Front Immunol. 2022 Jun 30;13:857934. doi: 10.3389/fimmu.2022.857934 (PMC9282908; doi:10.3389/fimmu.2022.857934)

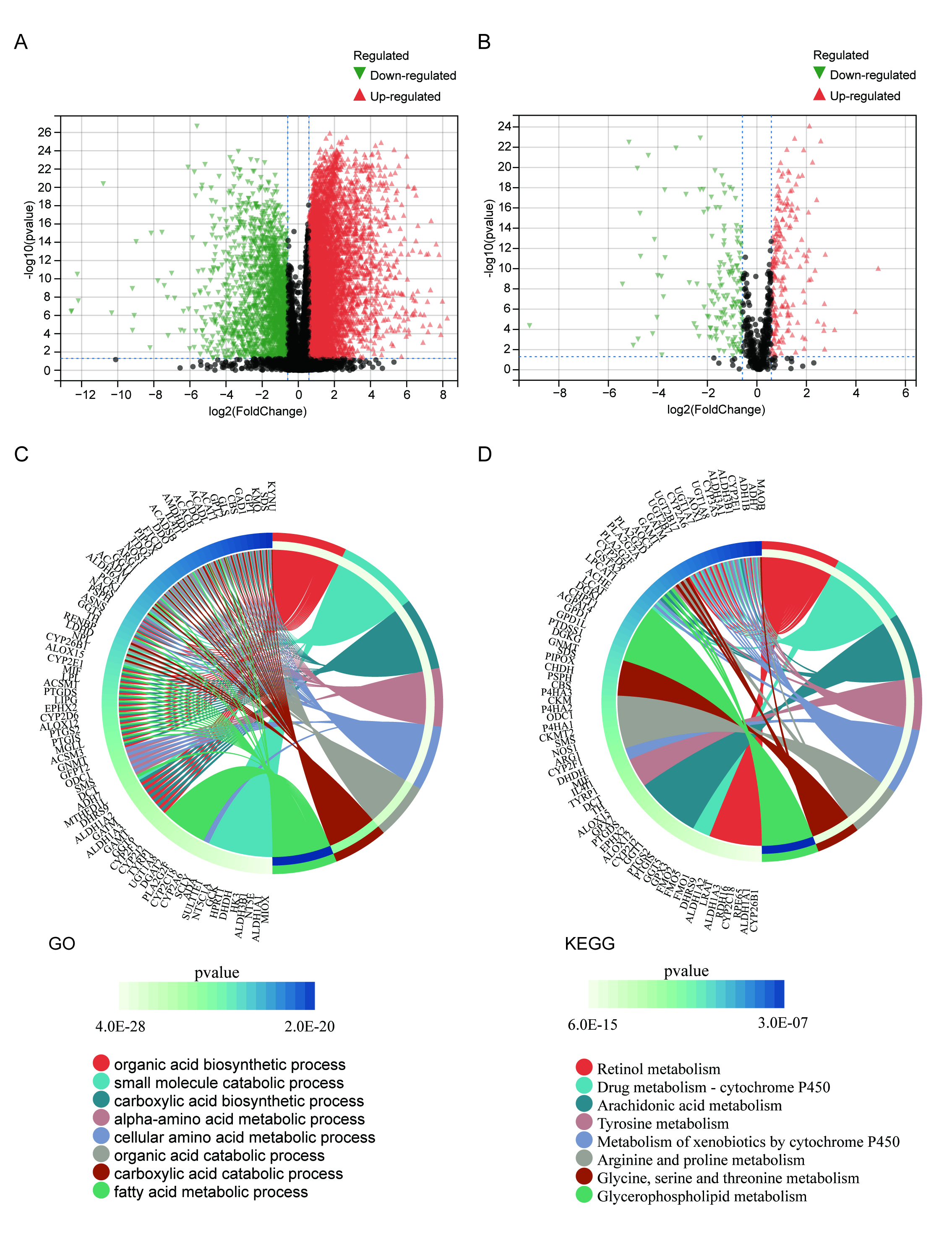

Supplement: Supplementary Figure 1 — Differentially expressed metabolism-related genes in HNSCC. (A) Volcano map exhibiting all differentially expressed genes (DEGs) between 502 HNSCC samples and 44 para-cancer samples (p < 0.05, |log2FC| > 0.585). (B) Volcano map displaying metabolism-related DEGs between 502 HNSCCC samples and 44 para-cancer samples. (C) Gene Ontology (GO) enrichment analysis of the metabolism-related DEGs (p < 0.05). (D) Kyoto Encyclopedia of Genes and Genomes (KEGG) pathway analysis of the metabolism-related DEGs (p < 0.05). [file Image_1.tif]

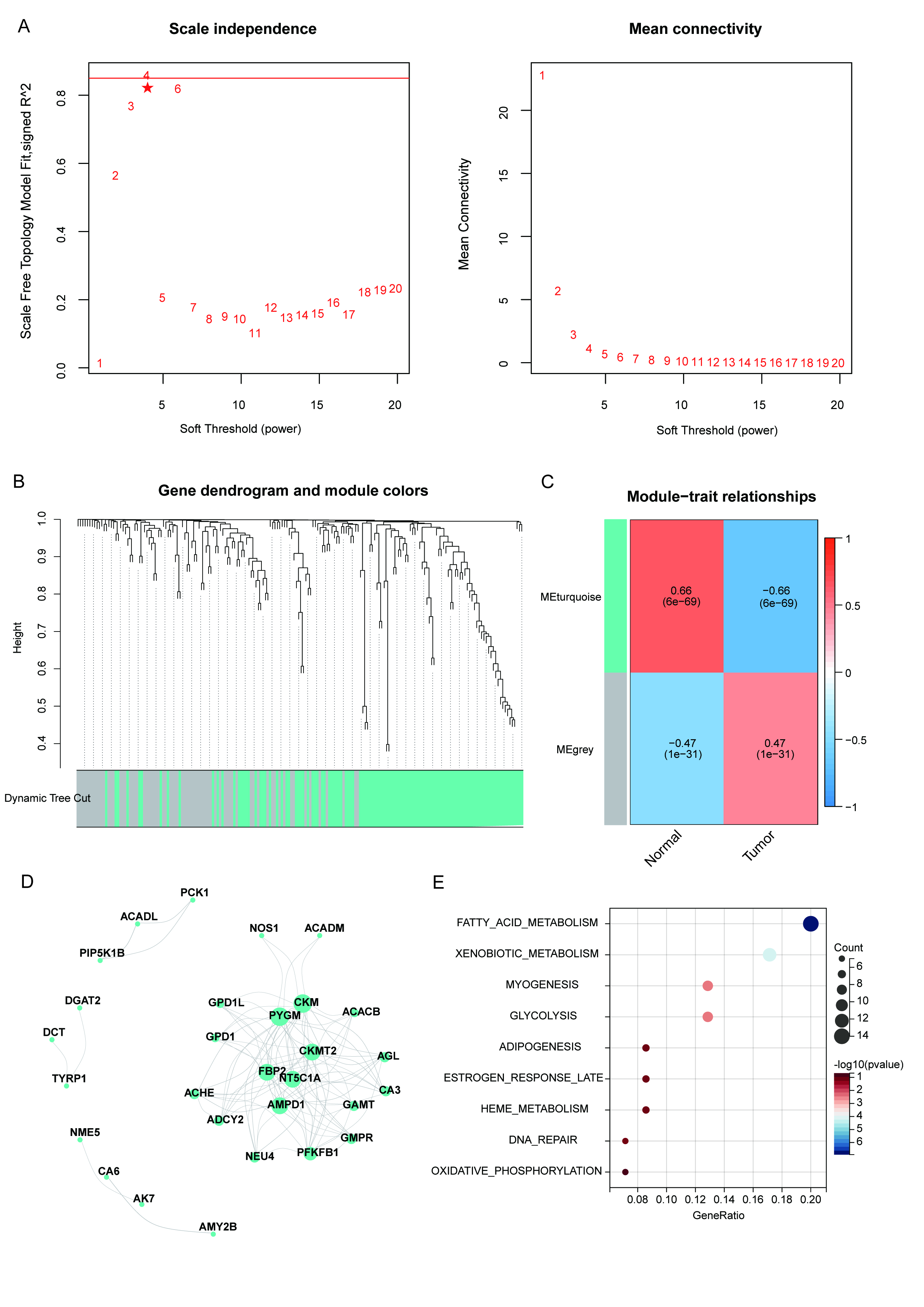

Supplement: Supplementary Figure 2 — Identification of metabolism-related hub genes by the WGCNA. (A) The soft-thresholding power was 0.85 determined by the red horizontal line in the Weighted gene coexpression network analysis (WGCNA) analysis and the optimal soft threshold for WGCNA was 4. (B) WGCNA of metabolism-related differentially expressed genes with a soft threshold β = 4. (C) Gene modules related to HNSCC obtained by WGCNA. (D) The network of the genes in the turquoise module (weight of edge > 0.2). (E) Hallmark enriched in the genes of the turquoise module (p < 0.05). [file Image_2.tif]

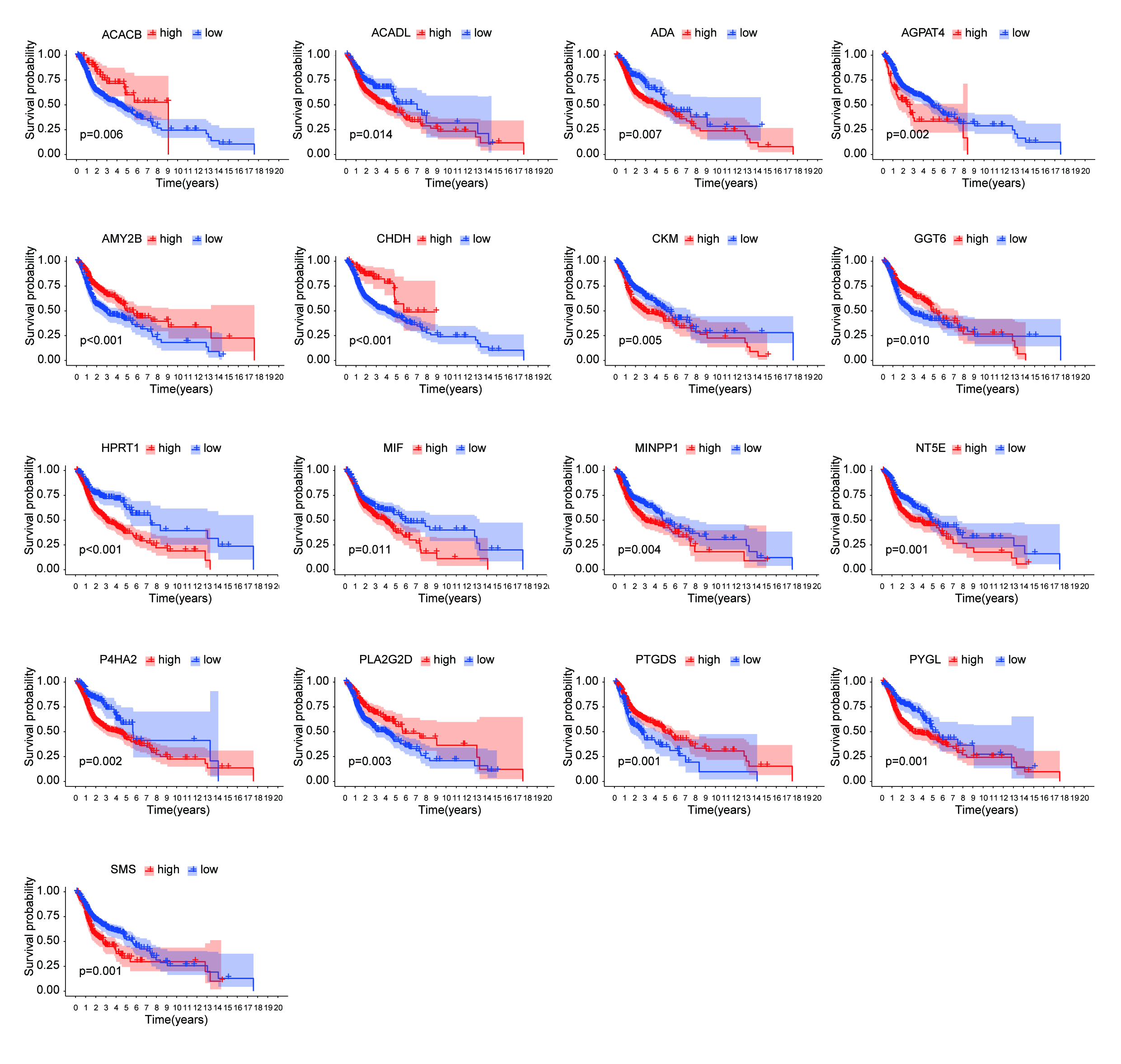

Supplement: Supplementary Figure 3 — Kaplan-Meier curves of 17 metabolism-related hub genes. Kaplan-Meier survival analysis of 17 metabolism-related genes in TCGA cohort. [file Image_3.tif]

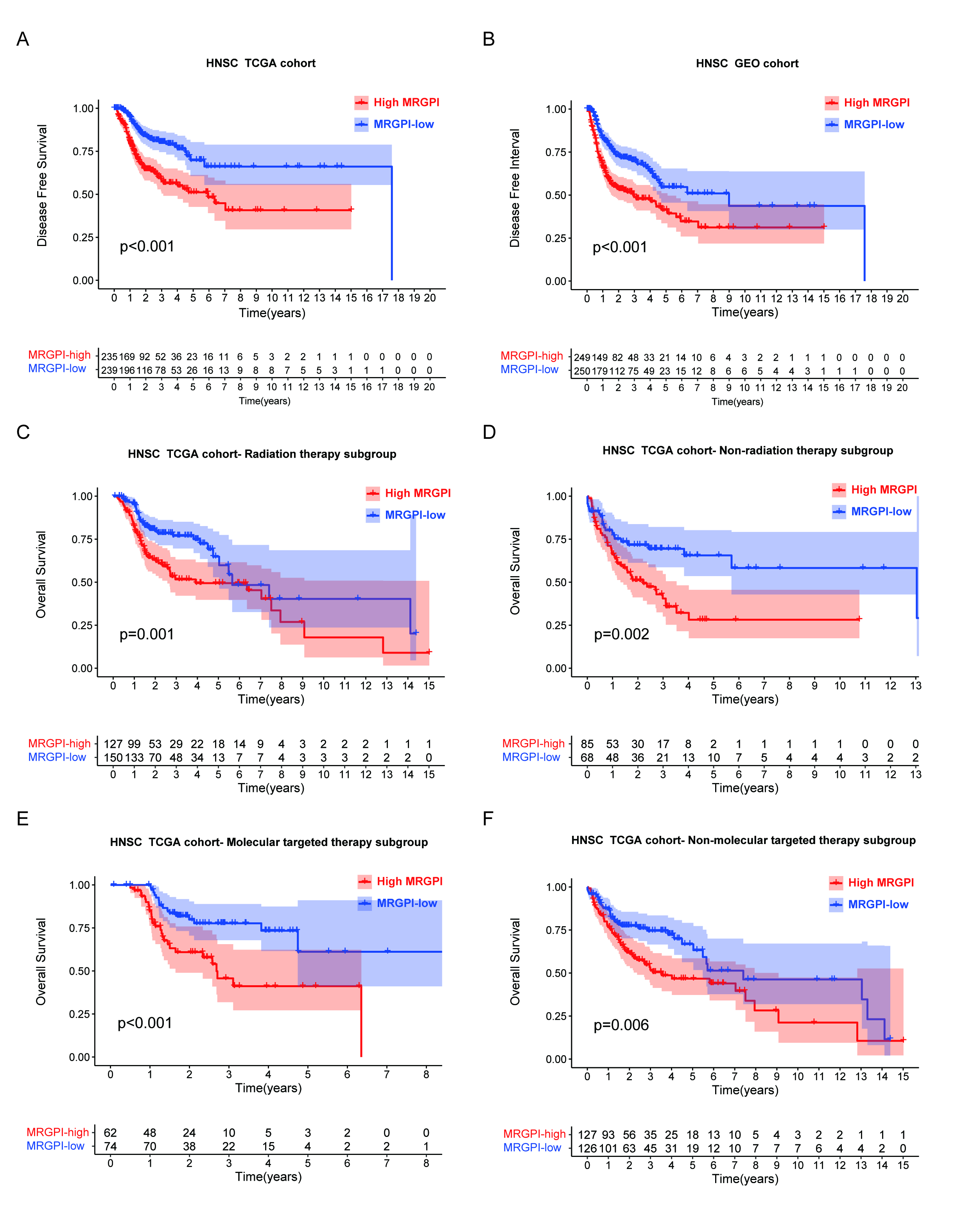

Supplement: Supplementary Figure 4 — The prognostic role of MRGPI. (A) Kaplan-Meier analysis of MRGPI subgroups on disease-free survival (DFS) in TCGA cohort. (B). Kaplan-Meier analysis of MRGPI subgroups on disease-free survival (DFS) in the GEO cohort. (C) Kaplan-Meier analysis of MRGPI subgroups for radiotherapy patients. (D) Kaplan-Meier analysis of MRGPI subgroups for non-radiotherapy patients. (E) Kaplan-Meier analysis of MRGPI subgroups for molecular targeted therapy patients. (F) Kaplan-Meier analysis of MRGPI subgroups for non-molecular targeted therapy patients. [file Image_4.tif]

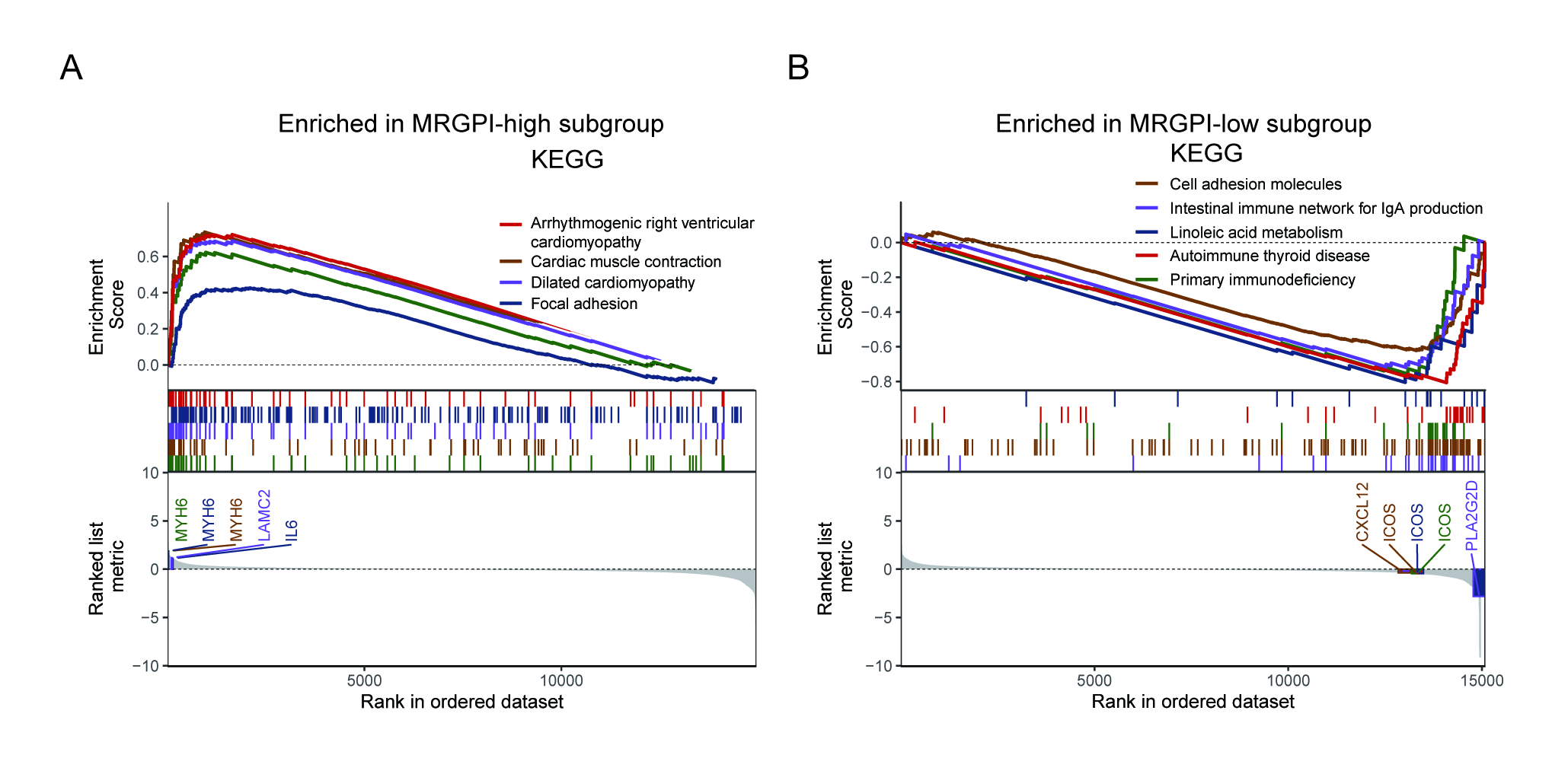

Supplement: Supplementary Figure 5 — Gene sets enriched in different MRGPI subgroups (A) KEGG gene sets enriched in MRGPI-high subgroup (P < 0.05, FDR < 0.25). (B) KEGG gene sets enriched in MRGPI-low subgroup (P < 0.05, FDR < 0.25). [file Image_5.tif]

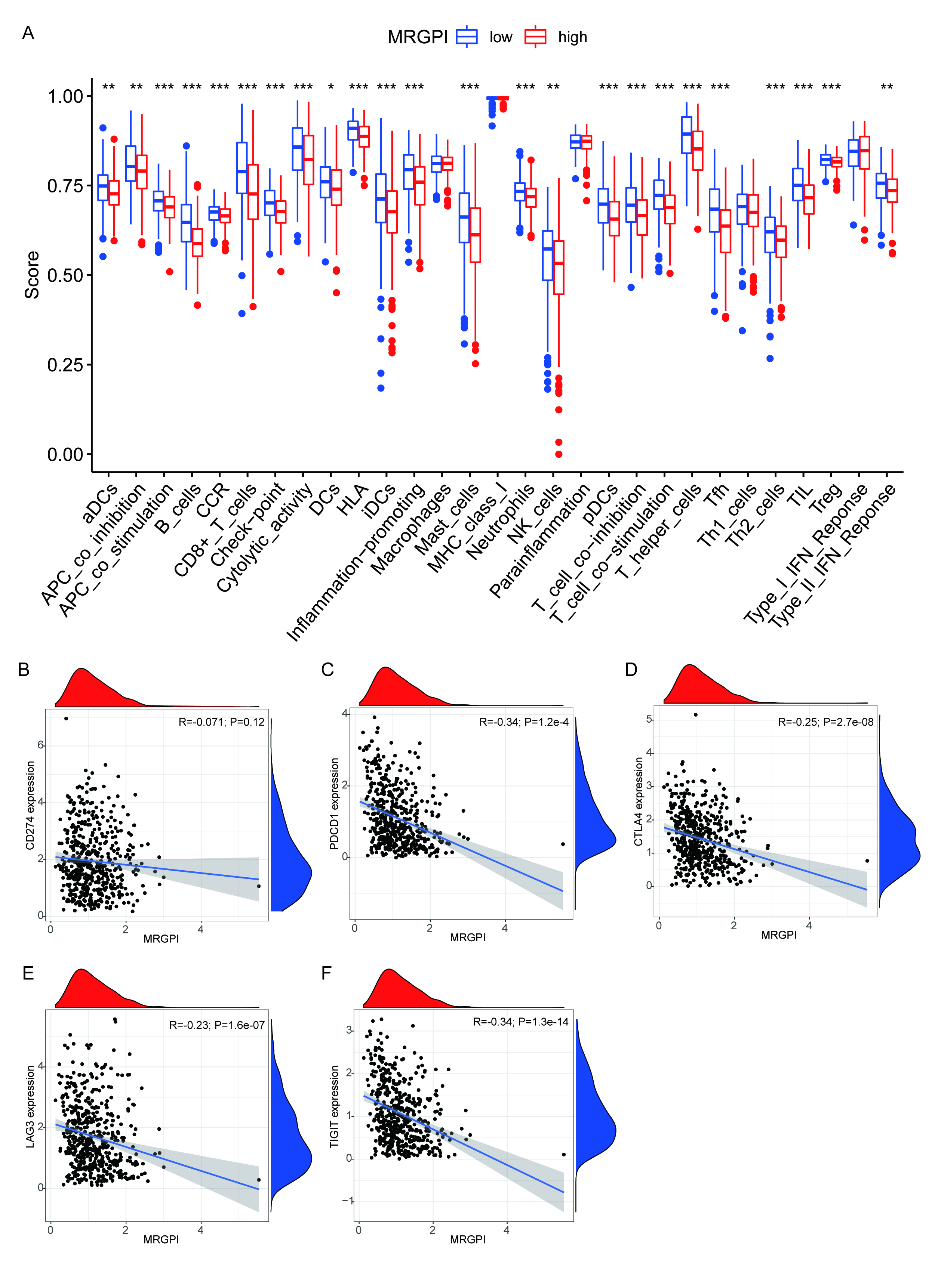

Supplement: Supplementary Figure 6 — The molecular and immune features of MRGPI. (A). The molecular and immune-related function of the different MRGPI subgroups. The gene sets of molecular and immune-related function were analyzed by the single sample gene set enrichment analysis (ssGSEA) and then compared between different MRGPI subgroups. The scattered dots represent the ssGSEA scores of the two subgroups. The thick lines represent the median value. The bottom and top of the boxes are the 25th and 75th percentiles (interquartile range), respectively. Significant statistical differences between the two subgroups were assessed using the Wilcoxon test. The relationship between MRGPI and immune checkpoint genes, such as (B) PD-L1, (C) PD-1, (D) CTLA-4, (E) LAG3, and (F) TIGIT. [file Image_6.tif]
