# Supplementary material for: A Metabolism-Related Gene Prognostic Index Bridging Metabolic Signatures and Antitumor Immune Cycling in Head and Neck Squamous Cell Carcinoma
Source: Front Immunol. 2022 Jun 30;13:857934. doi: 10.3389/fimmu.2022.857934 (PMC9282908; doi:10.3389/fimmu.2022.857934)
Supplement: Supplementary file 8 [file Table_8.docx]

| **Table S8. Correlation between MRGPI subgroups and clinicopathological factors in TCGA cohort** | | | |
| --- | --- | --- | --- |
| Variable | MRGPI-high  (n = 249) | MRGPI-low  (n = 250) | *P-*value |
| Age (Mean±SD) | 60.82±11.94 (53,68) | 61.34±11.92 (54,69) | 0.63 |
| Gender |  |  |  |
| Female | 62 | 71 | 0.43 |
| Male | 187 | 179 |  |
| Smoke history |  |  | 0.08 |
| No | 75 | 63 |  |
| Yes | 195 | 183 |  |
| Alcohol history |  |  | 0.53 |
| No | 80 | 88 |  |
| Yes | 169 | 162 |  |
| Grade |  |  | 0.21 |
| Grage1/2 | 189 | 170 |  |
| Grage3/4 | 55 | 66 |  |
| Tumor stage |  |  | 0.93 |
| Stage I/II | 55 | 58 |  |
| Stage III/IV | 185 | 187 |  |
| Neoadjuvant therapy |  |  | 0.33 |
| No | 242 | 247 |  |
| Yes | 7 | 3 |  |
| Radiotherapy |  |  | 0.08 |
| No | 75 | 63 |  |
| Yes | 113 | 141 |  |
| Survival status |  |  | <0.001 |
| Alive | 115 | 167 |  |
| Dead | 134 | 217 |  |
